# Supplementary material for: Seasonal pigment fluctuation in diploid and polyploid Arabidopsis revealed by machine learning-based phenotyping method PlantServation
Source: Nat Commun. 2023 Sep 22;14:5792. doi: 10.1038/s41467-023-41260-3 (PMC10517152; doi:10.1038/s41467-023-41260-3)
Supplement: Supplementary file 1 — Supplementary Information [file 41467_2023_41260_MOESM1_ESM.pdf]

Supplementary Information

Seasonal pigment fluctuation in diploid and polyploid *Arabidopsis* revealed by machine learning-based phenotyping method PlantServation

Reiko Akiyama[1†], Takao Goto[2†], Toshiaki Tameshige[3,4†], Jiro Sugisaka[3,5], Ken Kuroki[6], Jianqiang Sun[7], Junichi Akita[8], Masaomi Hatakeyama[1,9], Hiroshi Kudoh[5], Tanaka Kenta[10], Aya Tonouchi[2], Yuki Shimahara[2], Jun Sese[11,12,13], Natsumaro Kutsuna[2], Rie Shimizu-Inatsugi[1*], Kentaro K. Shimizu[1,3*]

[1] Department of Evolutionary Biology and Environmental Studies, University of Zurich, Winterthurerstrasse 190, CH-8057, Zurich, Switzerland
[2] Research and Development Division, LPIXEL Inc., Chiyoda-ku, Tokyo 100–0004, Japan
[3] Kihara Institute for Biological Research (KIBR), Yokohama City University, 641-12 Maioka, Totsuka-ward, Yokohama, 244-0813, Japan
[4] Division of Biological Science, Graduate School of Science and Technology, Nara Institute of Science and Technology (NAIST), 8916-5 Takayama-Cho, Ikoma, Nara 630-0192, Japan
[5] Center for Ecological Research, Kyoto University, Hirano 2-509-3, Otsu 520-2113, Japan
[6] Department of Biological Sciences, Graduate School of Science, The University of Tokyo, 7-3-1 Hongo, Bunkyo-ku, Tokyo 113-0033, Japan
[7] Research Center for Agricultural Information Technology, National Agriculture and Food Research Organization, 3-1-1 Kannondai, Tsukuba, Ibaraki, 305-8517, Japan
[8] Department of Electric and Computer Engineering, Kanazawa University, Kakuma, Kanazawa 920-1192, Japan
[9] Functional Genomics Center Zurich, Winterthurerstrasse 190, CH-8057, Zurich, Switzerland
[10] Sugadaira Research Station, Mountain Science Center, University of Tsukuba, 1278-294 Sugadaira-kogen, Ueda 386-2204, Japan
[11] Artificial Intelligence Research Center, AIST, 2-3-26 Aomi, Koto-ku, Tokyo 135-0064, Japan
[12] Humanome Lab, Inc., L-HUB 3F, 1-4, Shumomiyabi-cho, Shinjuku, Tokyo, 162-0822, Japan
[13] AIST-Tokyo Tech RWBC-OIL, 2-12-1 O-okayama, Meguro-ku, Tokyo 152-8550, Japan

† These authors contributed equally
* Corresponding authors
Kentaro K. Shimizu: kentaro.shimizu@uzh.ch; Rie Shimizu-Inatsugi: rie.inatsugi@ieu.uzh.ch

This file includes:
Supplementary Figs 1 to 31
Supplementary Tables 1 to 9

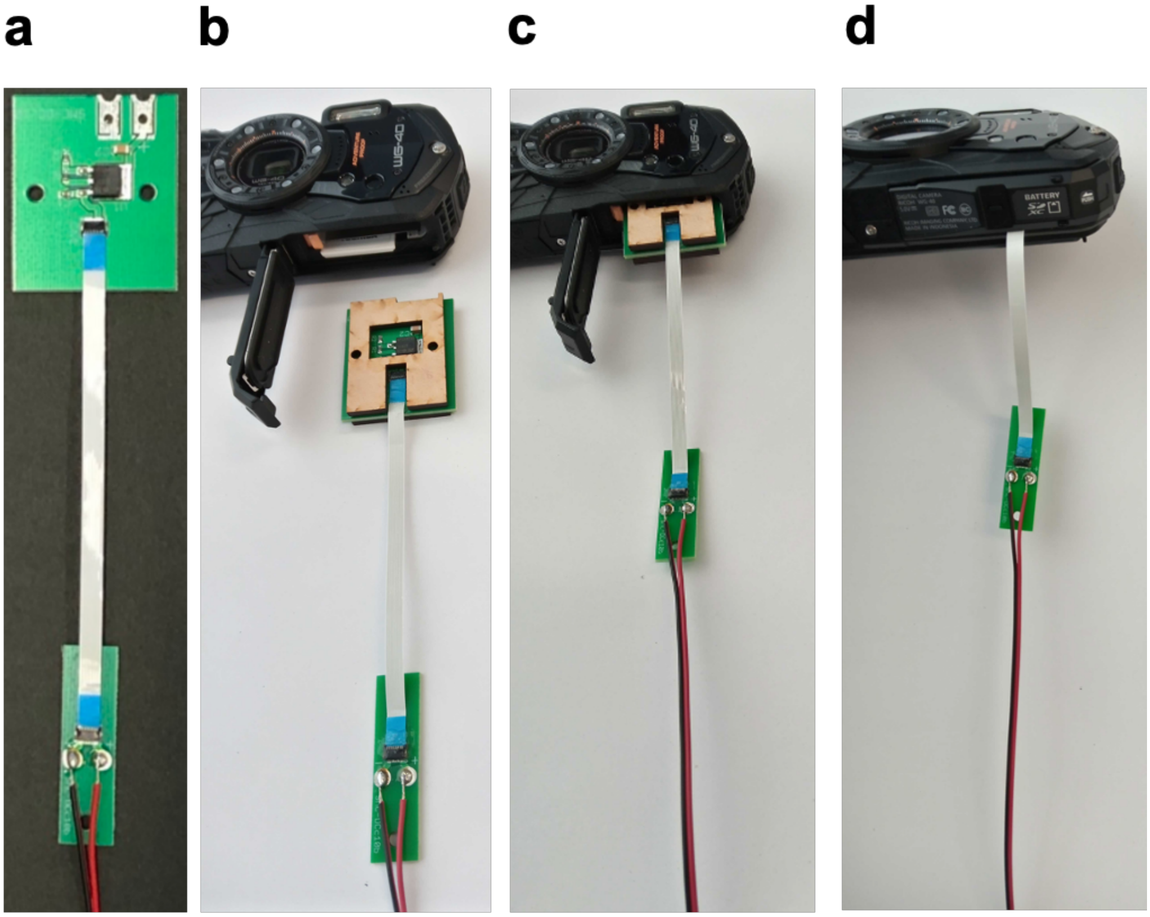

Supplementary Fig. 1. Details of the set-up consisting of a DC coupler, wooden fixing panels, a flat cable, and a connector. **(a)** The set-up without wooden fixing panels where the DC coupler is visible. **(b)** The set-up and a camera. **(c)** The DC coupler and wooden fixing panels are half-inserted in the battery slot of a camera. **(d)** The DC coupler and wooden fixing panels are in the battery slot, while the flat cable comes out with the battery lid closed. The blueprint of the DC coupler is available at: https://github.com/akita11/RicohCameraDCCoupler

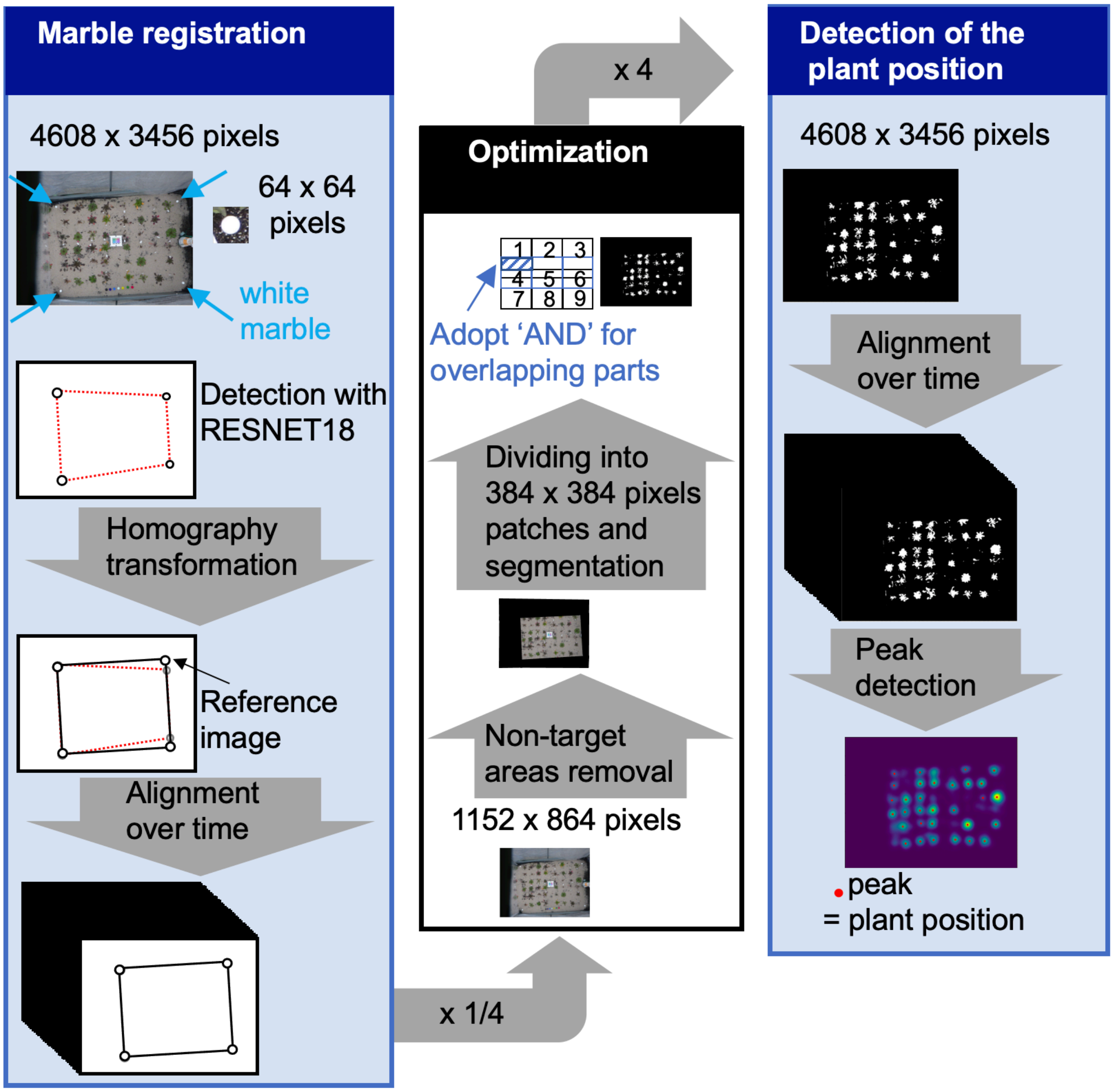

Supplementary Fig. 2. The workflow of the detection of the plant position. Marble registration was conducted first to align the position of the images over time. Optimization was performed to reduce the processing time. The plant position was defined as the peak detected in the aligned images.

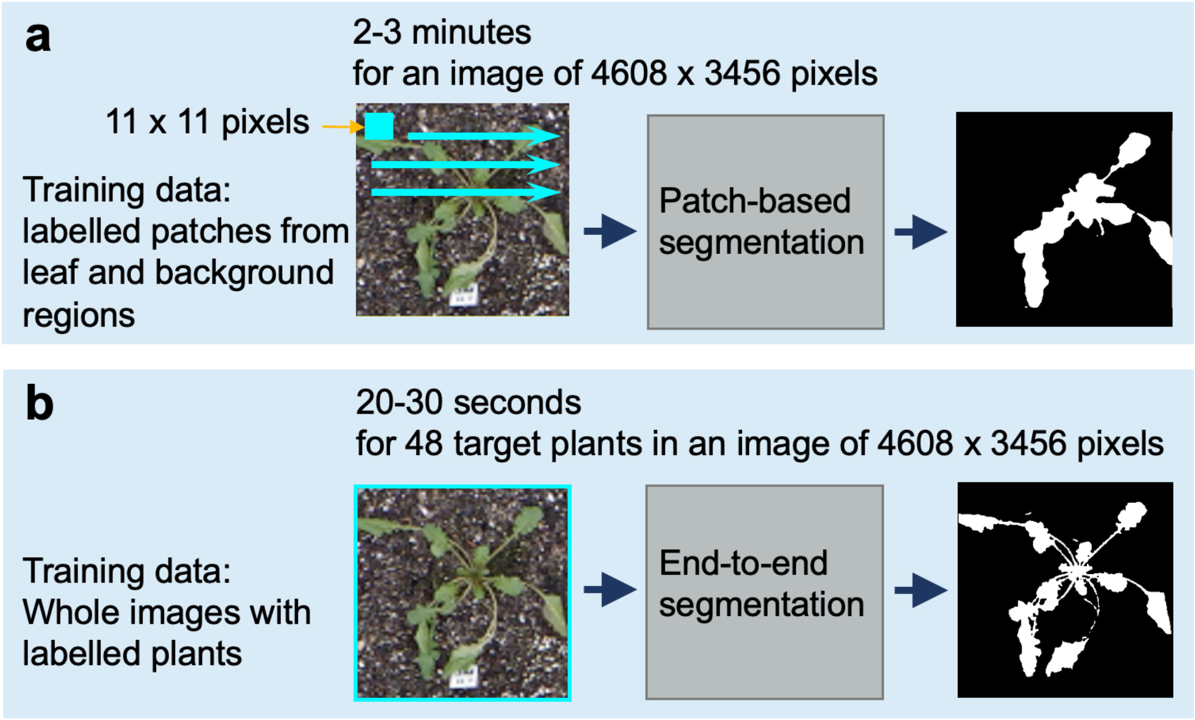

Supplementary Fig. 3. Workflows of a patch-based and an end-to-end segmentation. **(a)** Patch-based method: whether the pixel in the center of a patch of 11 x 11 pixels is a leaf or not was determined. This procedure was repeated for all the 4608 x 3456 pixels in the image. **(b)** End-to-end method (DANet): an area including a target plant was used as an input.

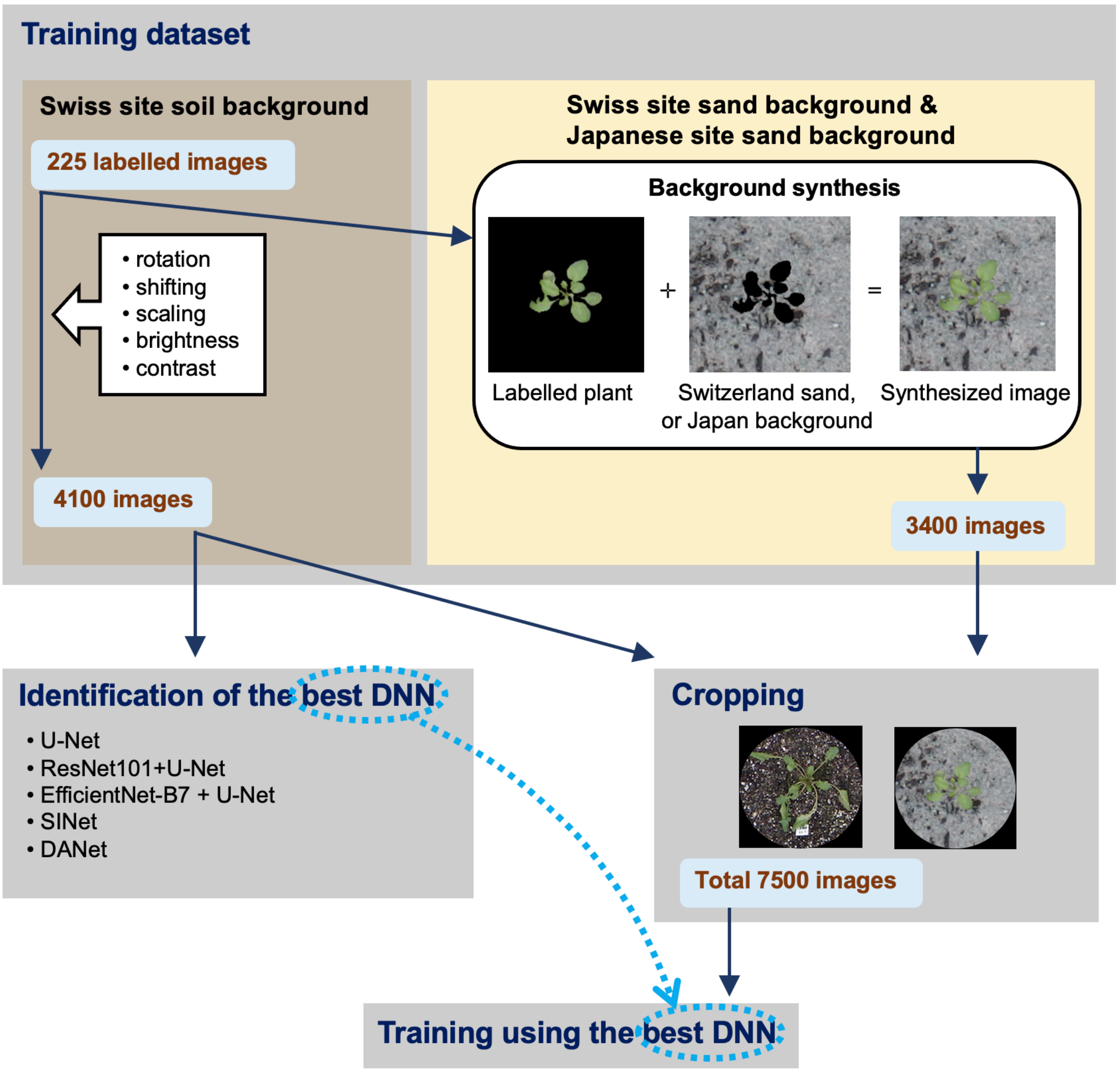

Supplementary Fig. 4. Overview of the training of the plant segmentation model, consisting of the preparation of the training dataset, identification of the best DNN for the study dataset, and cropping of the images for training. Processes with white backgrounds correspond to augmentation.

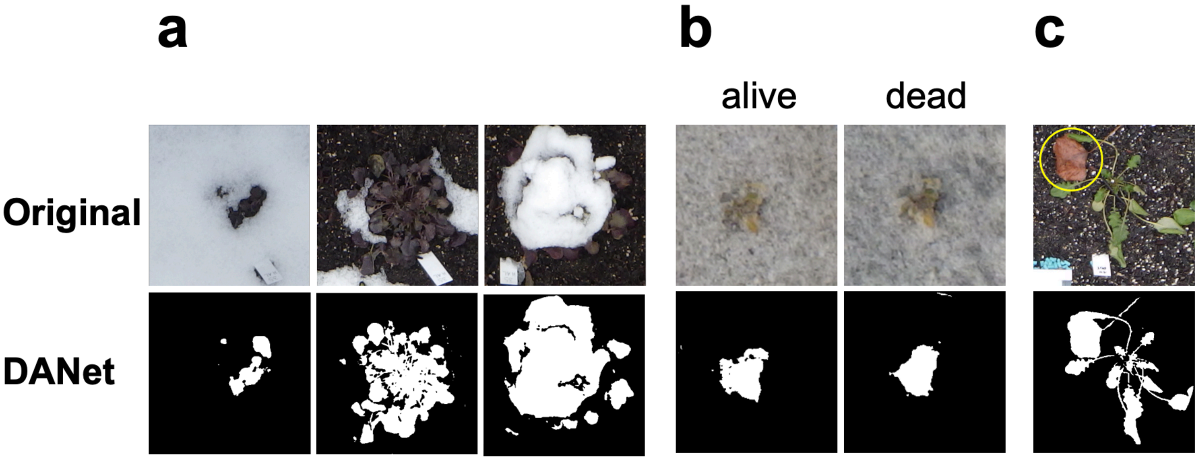

Supplementary Fig. 5. Examples of factors complicating the interpretation of the segmentation outcome. **(a)** Snow. **(b)** Plant death. **(c)** Fallen leaf (in yellow circle). In **(a)**-**(c)**, the original image and the outcome of the segmentation by DANet are shown in the top and bottom rows, respectively.

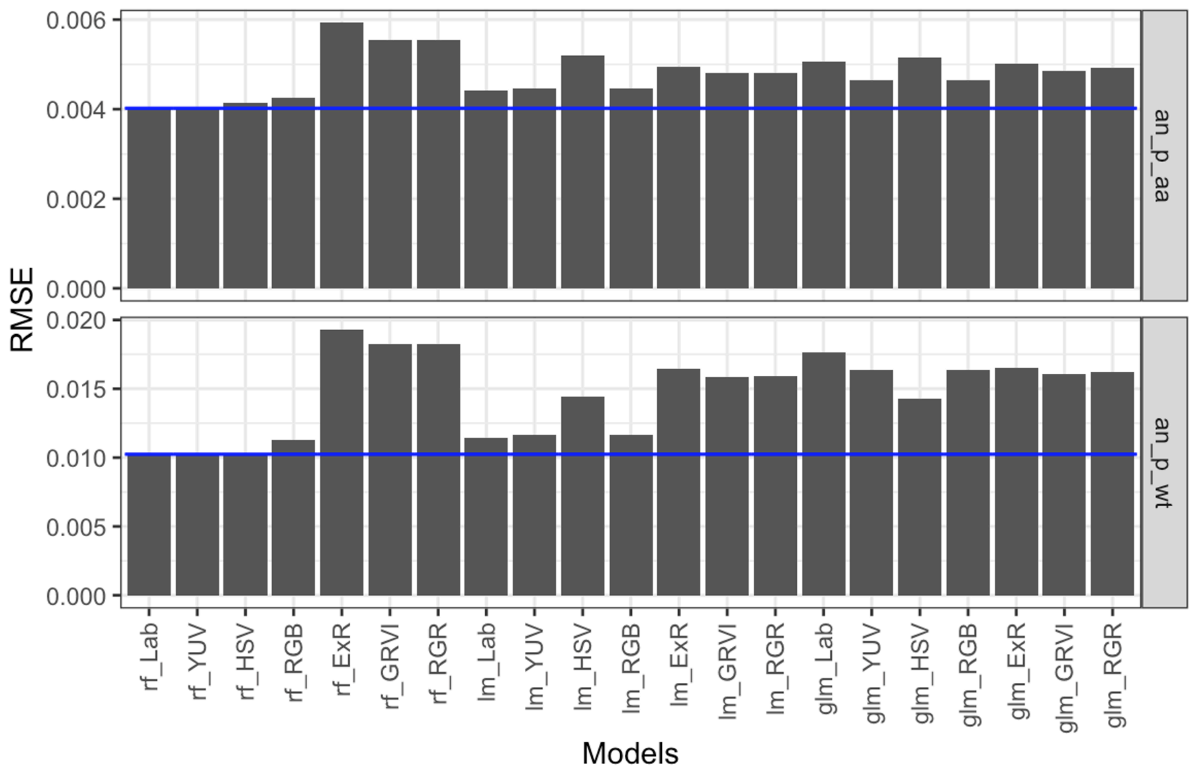

Supplementary Fig. 6. Bar charts of root mean square error (RMSE) for measured and estimated values for different models for two response variables (an_p_aa: anthocyanin per area, an_p_wt: anthocyanin per weight) for plants of 12 *Arabidopsis* genotypes. Blue horizontal lines indicate the smallest RMSE for each response variable. For each model, letters before _ indicate the model type (rf: random forest, lm: linear model, glm: generalized linear model) while letters after _ indicate the explanatory variable (Lab: L* + a* + b*, YUV: Y + U + V, HSV: H + S + V, RGB: R + G + B, ExR: Excess Red, GRVI: Green-Red Vegetation Index, RGR: Red Green Ratio). Source data are provided as a Source Data file.

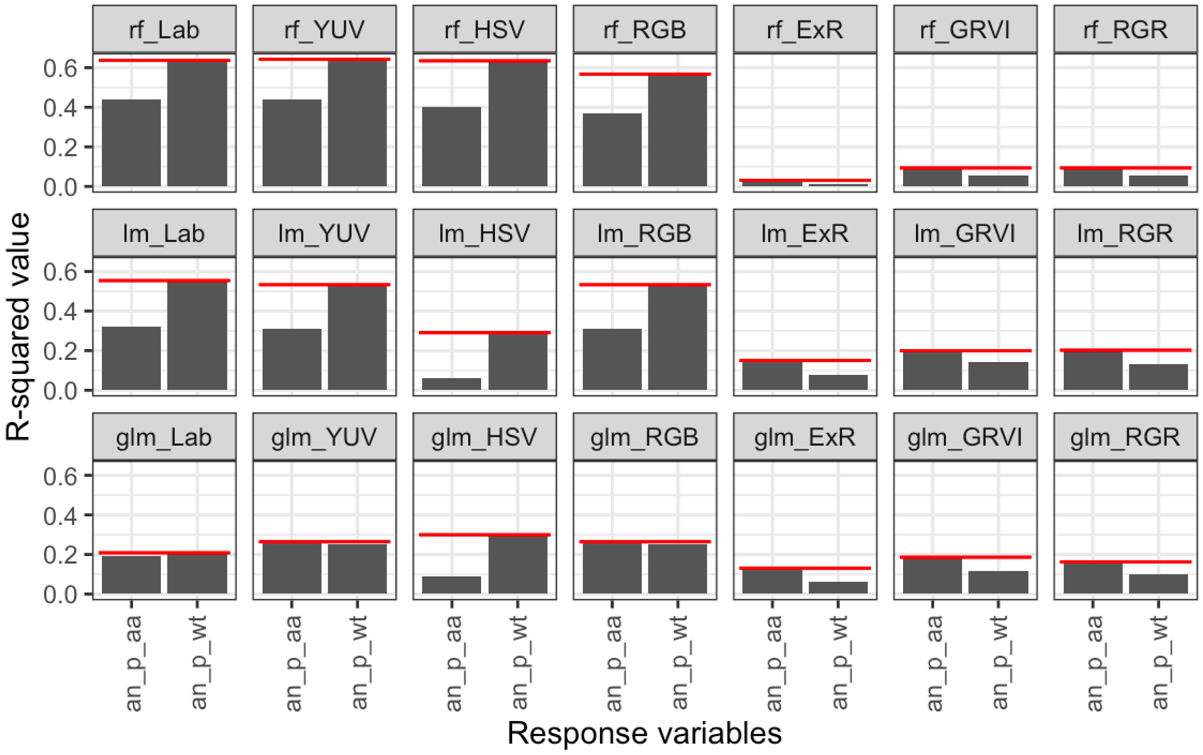

Supplementary Fig. 7. Bar charts of R-squared value (R^2) for measured and estimated values for different response variables (an_p_aa: anthocyanin per area, an_p_wt: anthocyanin per weight) for plants of 12 *Arabidopsis* genotypes for each of the models. For each model, letters before _ indicate the model type (rf: random forest, lm: linear model, glm: generalized linear model) while letters after _ indicate the explanatory variable (Lab: L* + a* + b*, YUV: Y + U + V, HSV: H + S + V, RGB: R + G + B, ExR: Excess Red, GRVI: Green-Red Vegetation Index, RGR: Red Green Ratio). Red horizontal lines indicate the largest R^2 value for each model. Source data are provided as a Source Data file.

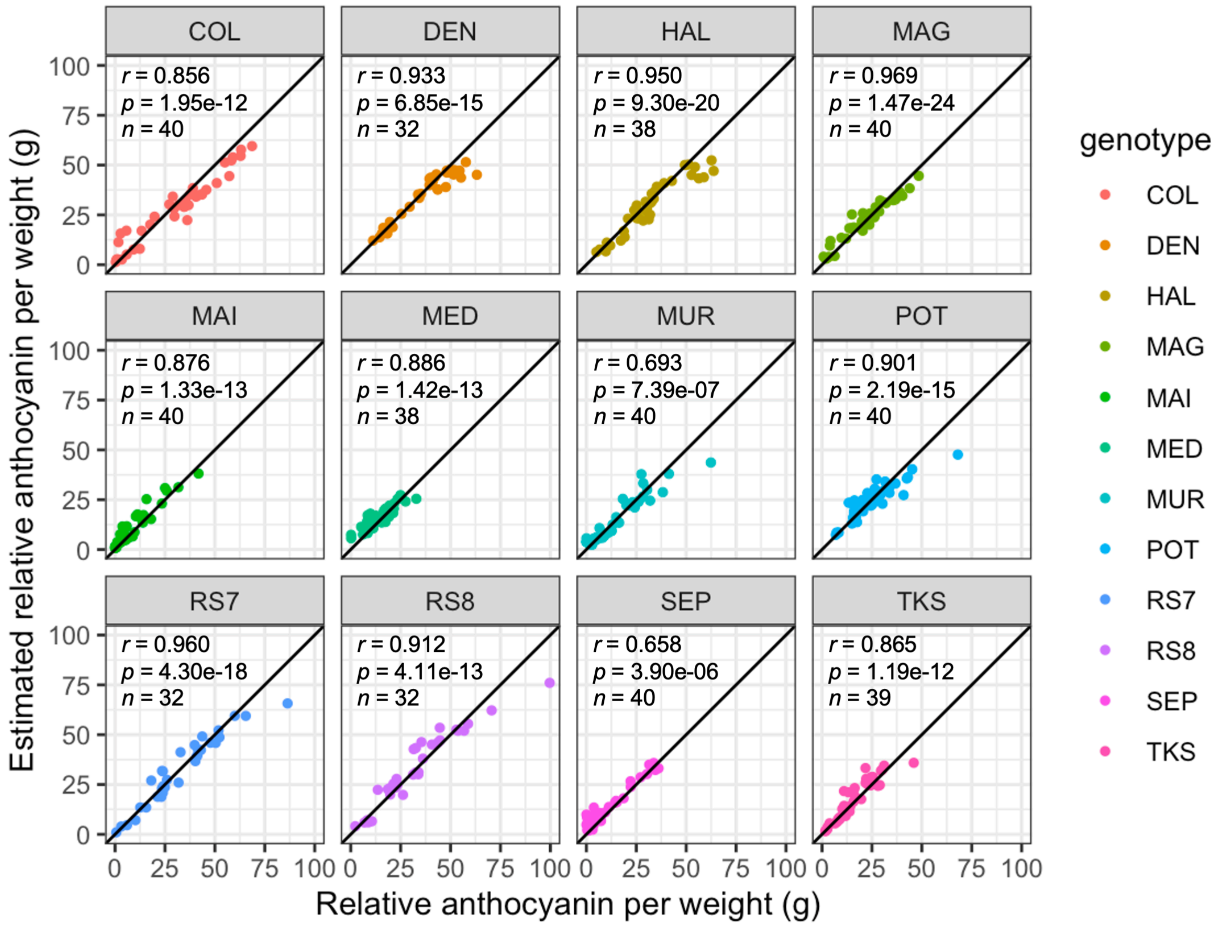

Supplementary Fig. 8. The results of fitting of the random forest model to estimate the relative anthocyanin per weight of leaves for each genotype of *Arabidopsis*. The scatter plots show the correlation between the relative anthocyanin per weight and the estimated relative anthocyanin per weight using the random forest model. The correlation coefficient (r) and p-value from Pearson's two-sided correlation tests are indicated in the plots. Source data are provided as a Source Data file.

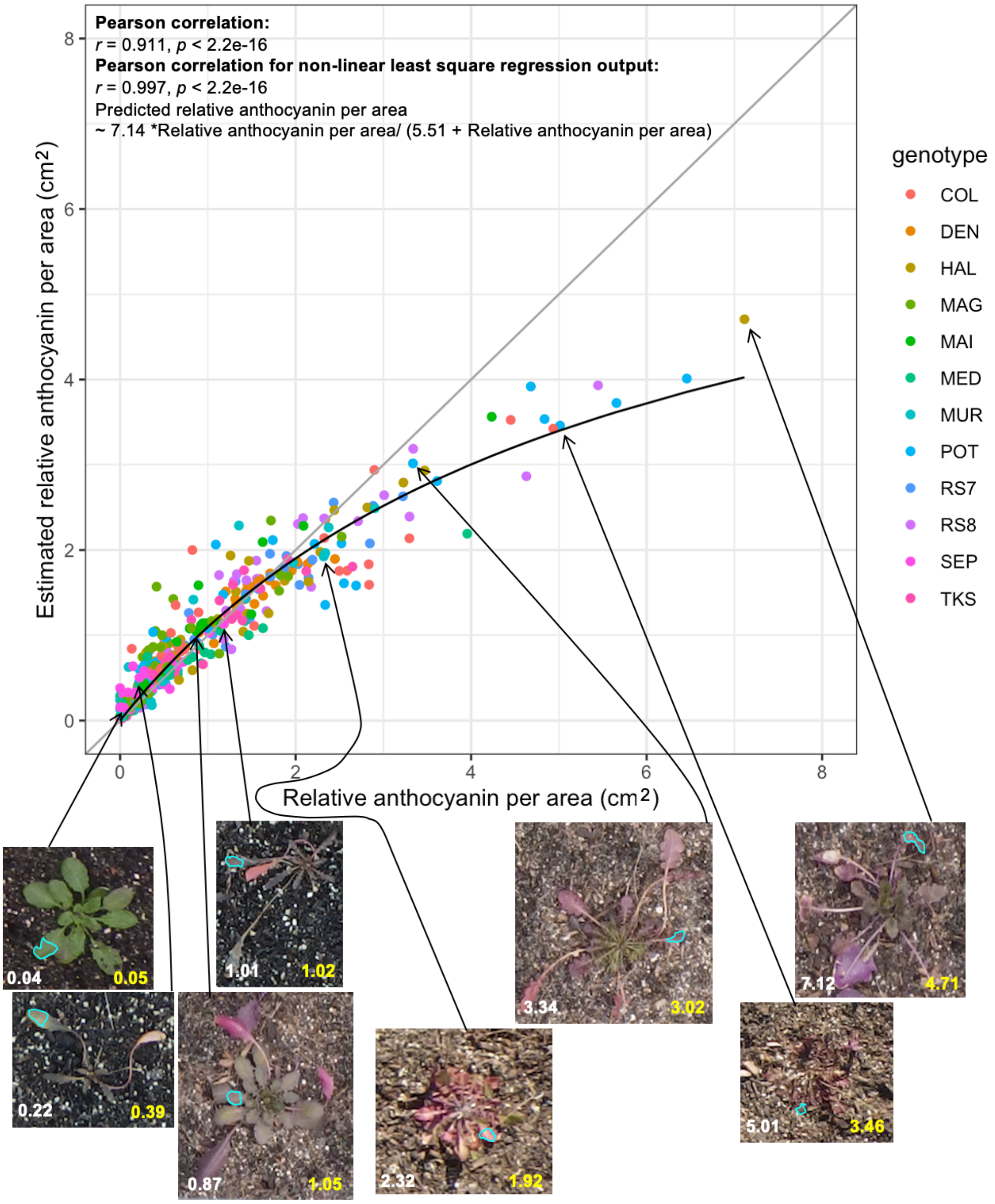

Supplementary Fig. 9. Scatter plot of relative anthocyanin content per area and estimated relative anthocyanin content per area of leaves for 12 *Arabidopsis* genotypes using random forest model. The gray line indicates y = x, while the black line indicates a non-linear least square regression. The correlation coefficient (r) and p-value from Pearson's two-sided correlation tests between relative anthocyanin per area and relative anthocyanin per area estimated using a random forest model, and between relative anthocyanin per area and relative anthocyanin per area estimated using a non-linear least square regression model are indicated in the plot. n = 451. In the time-series image analysis, the whole plant area was analyzed using the random forest model built here. Images below the plot show representative examples of the estimation of the anthocyanin content using a random forest model. White and yellow numbers indicate measured and estimated values, respectively. The leaf area subject to the analysis is surrounded by cyan. Source data are provided as a Source Data file.

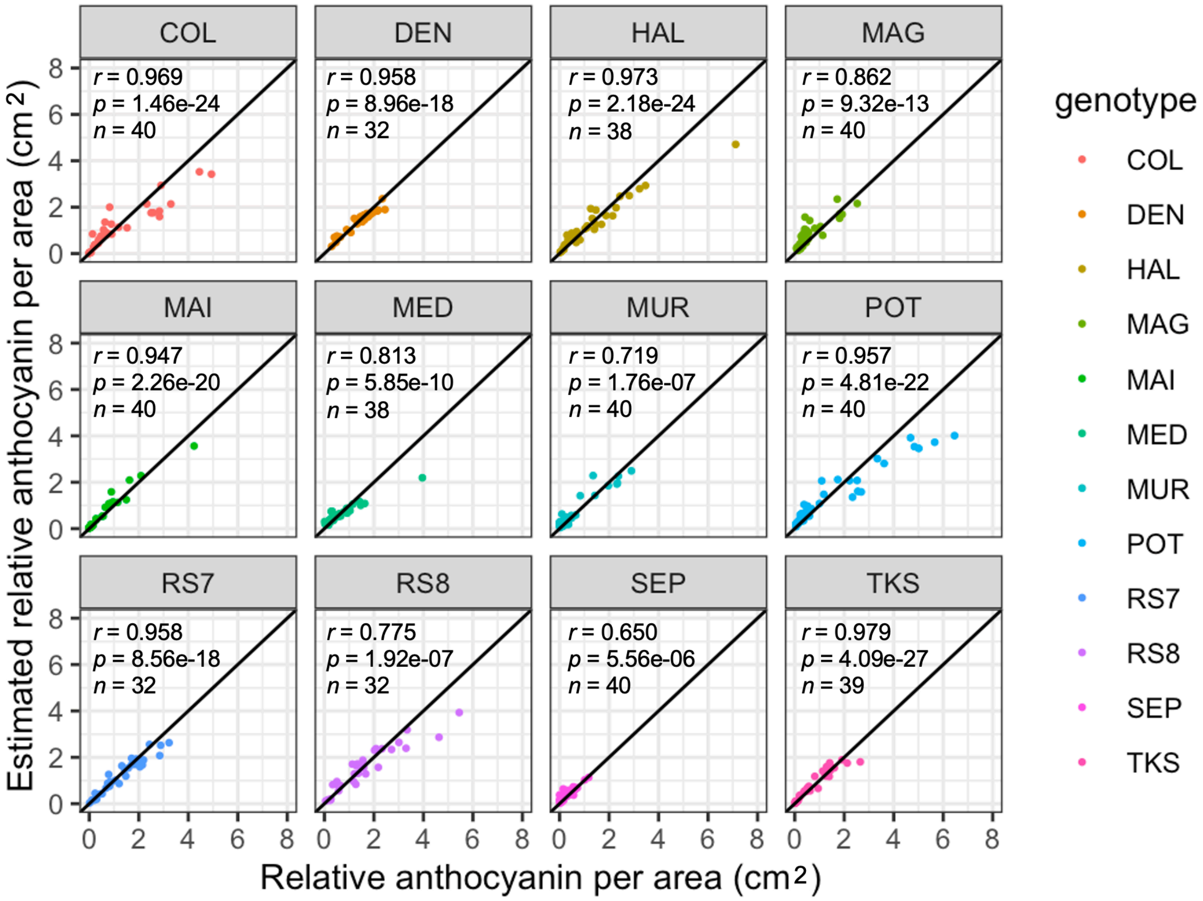

Supplementary Fig. 10. The results of fitting of the random forest model to estimate the relative anthocyanin per area of leaves for each genotype of *Arabidopsis*. The scatter plots show the correlation between the relative anthocyanin per area and the estimated relative anthocyanin per area using the random forest model. The correlation coefficient (r) and p-value from Pearson's two-sided correlation tests are indicated in the plots. Source data are provided as a Source Data file.

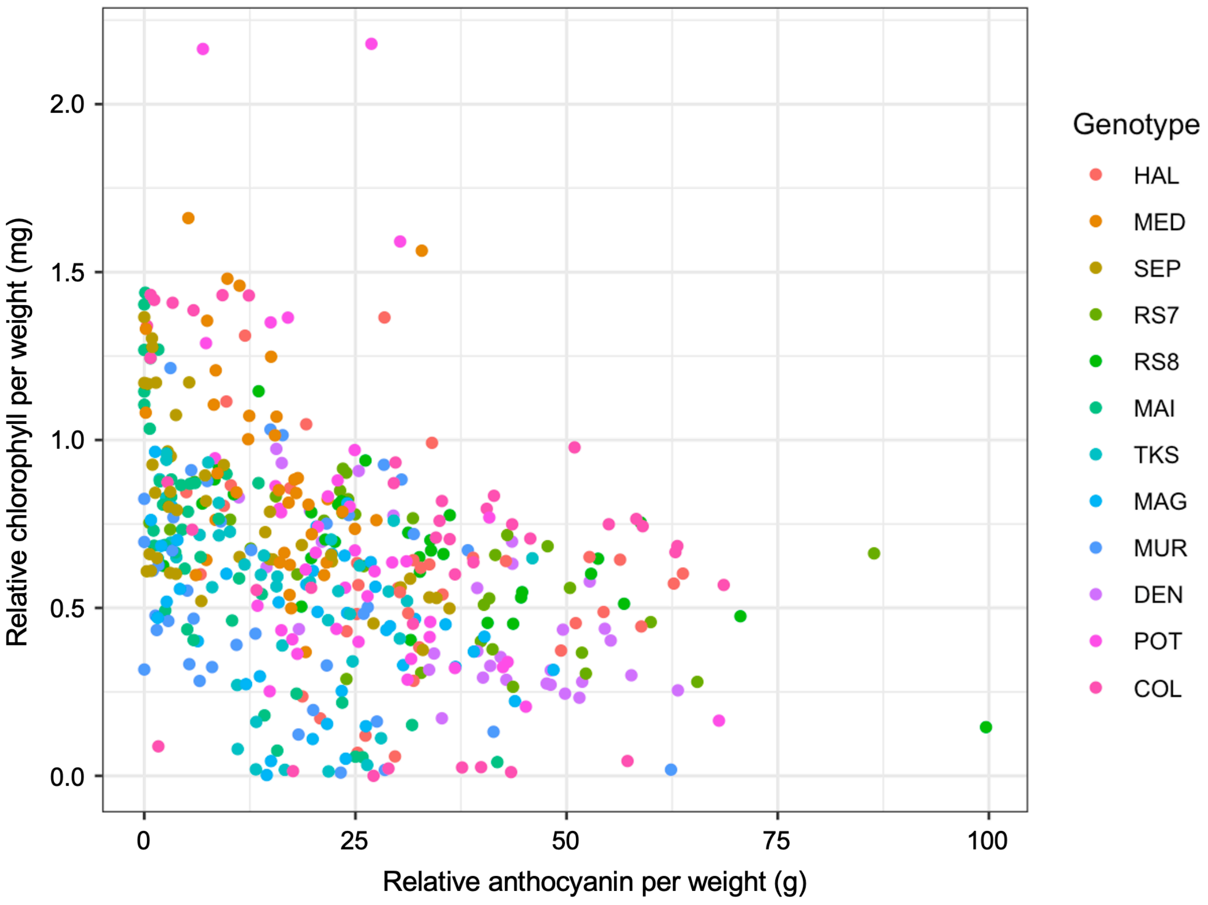

Supplementary Fig. 11. Scatter plot of relative anthocyanin content per weight and relative chlorophyll content per weight measured for 12 *Arabidopsis* genotypes. Data points are colored according to the genotype. Pearson's two-sided correlation test: $r = -0.433$, $p < 2.2e\text{-}16$. $n = 451$. Source data are provided as a Source Data file.

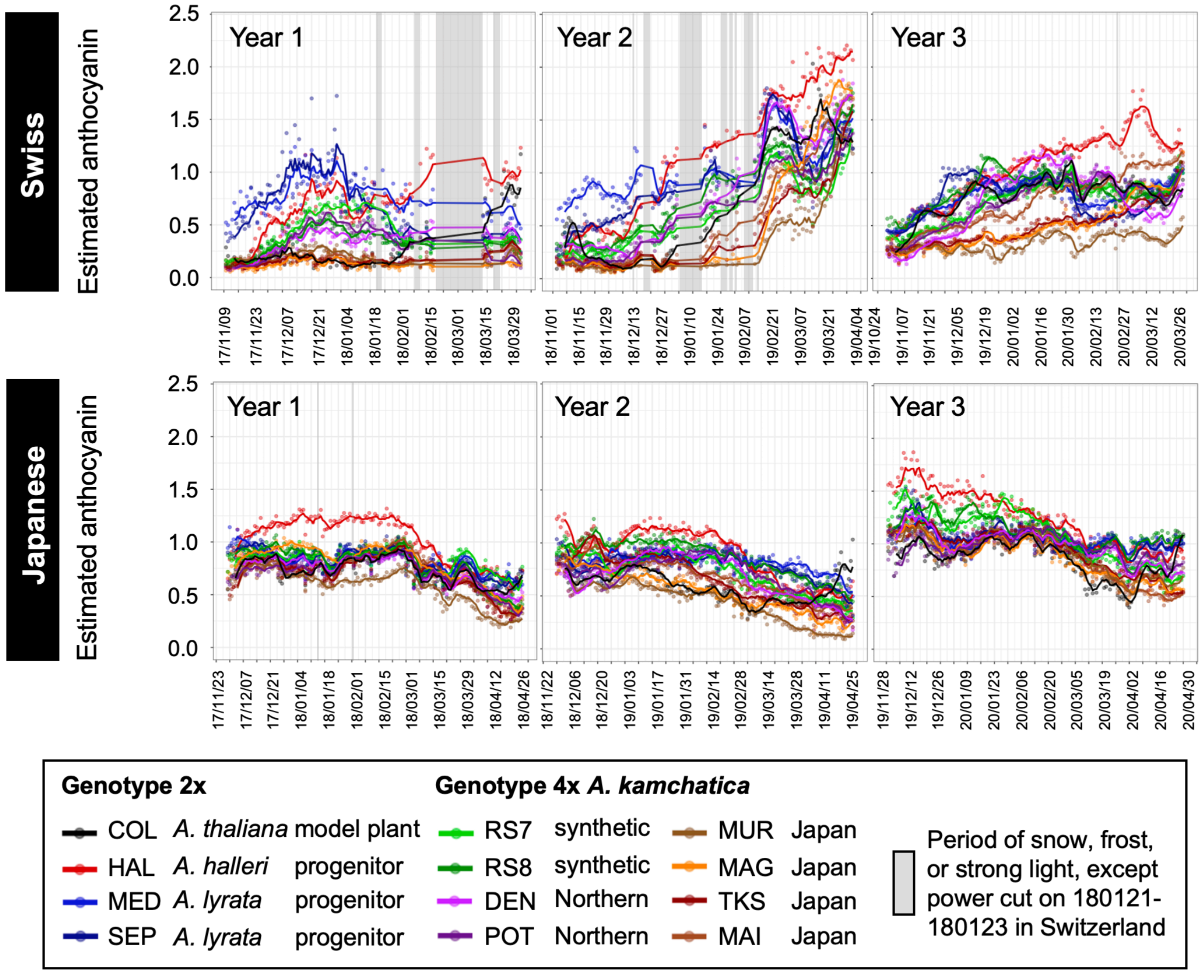

Supplementary Fig. 12. Time-series plots of 5-day moving average of estimated relative anthocyanin content per area (cm^2) in plants of 12 *Arabidopsis* genotypes at the Swiss and Japanese sites in three seasons. The data points indicate the arithmetic mean. The number of plants is summarized in Supplementary Table 8. Source data are provided as a Source Data file.

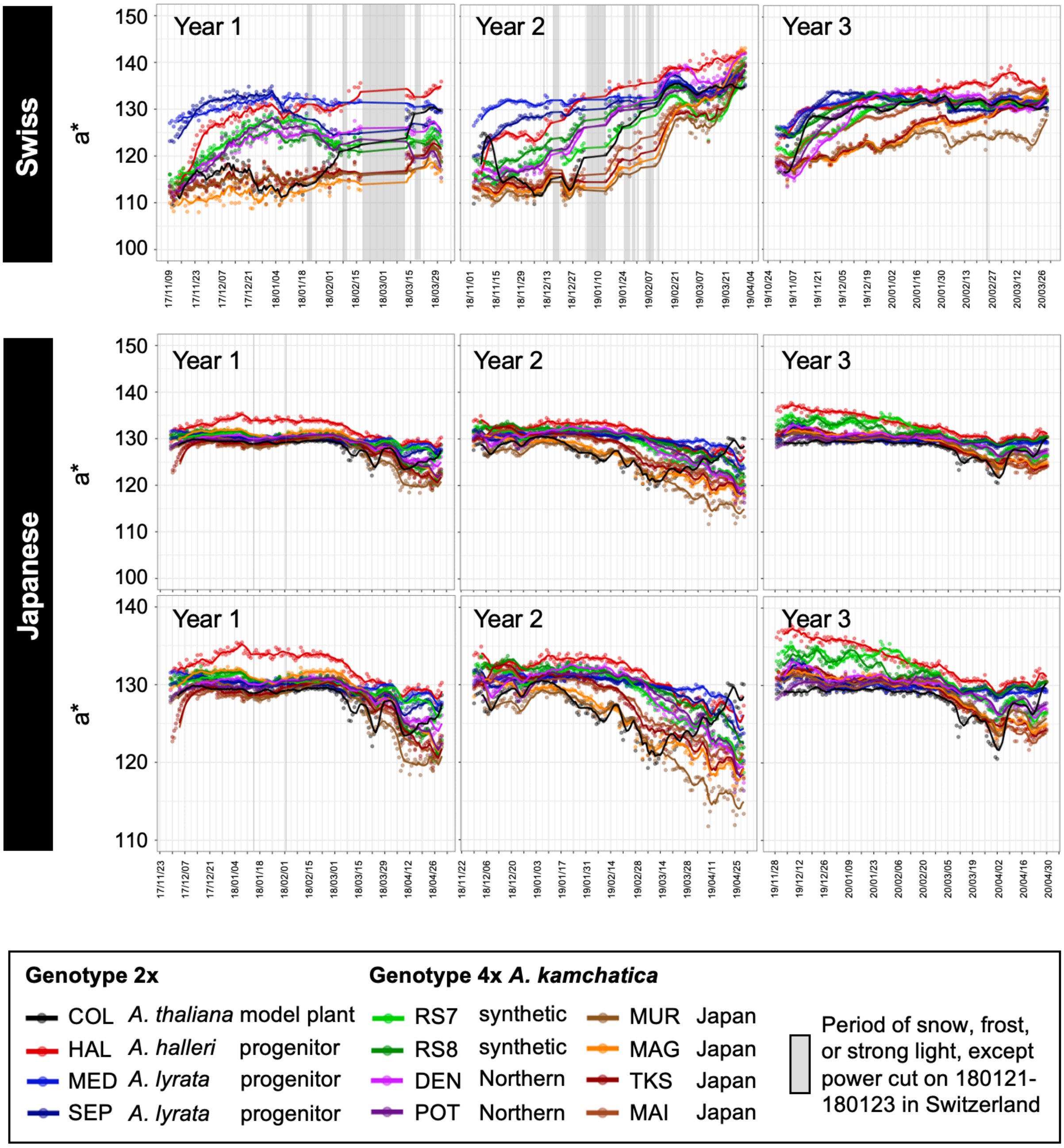

Supplementary Fig. 13. Time-series plots of the 5-day moving average of a* in plants of 12 *Arabidopsis* genotypes at the Swiss and Japanese sites in three seasons. The plots of the Japanese site are shown in two versions, where the top row presents the same Y-axis scale as that of the plots of the Swiss site and the bottom row presents the plots with an enlarged Y-axis for better visibility. The data points indicate the arithmetic mean. The number of plants is summarized in Supplementary Table 8. Source data are provided as a Source Data file.

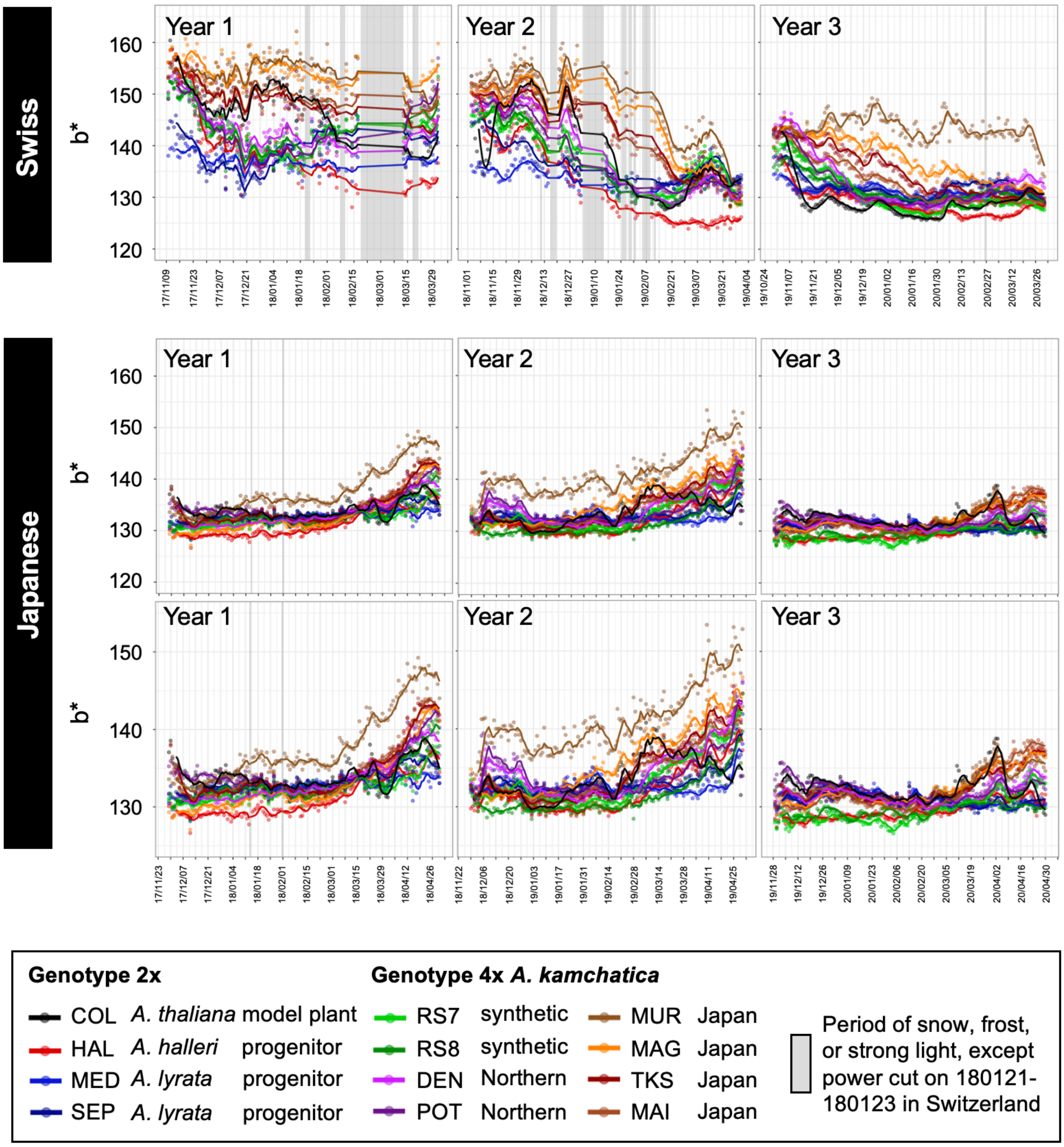

Supplementary Fig. 14. Time-series plots of the 5-day moving average of b* in plants of 12 *Arabidopsis* genotypes at the Swiss and Japanese sites in three seasons. The plots of the Japanese site are shown in two versions, where the top row presents the same Y-axis scale as that of the plots of the Swiss site and the bottom row presents the plots with an enlarged Y-axis for better visibility. The data points indicate the arithmetic mean. The number of plants is summarized in Supplementary Table 8. Source data are provided as a Source Data file.

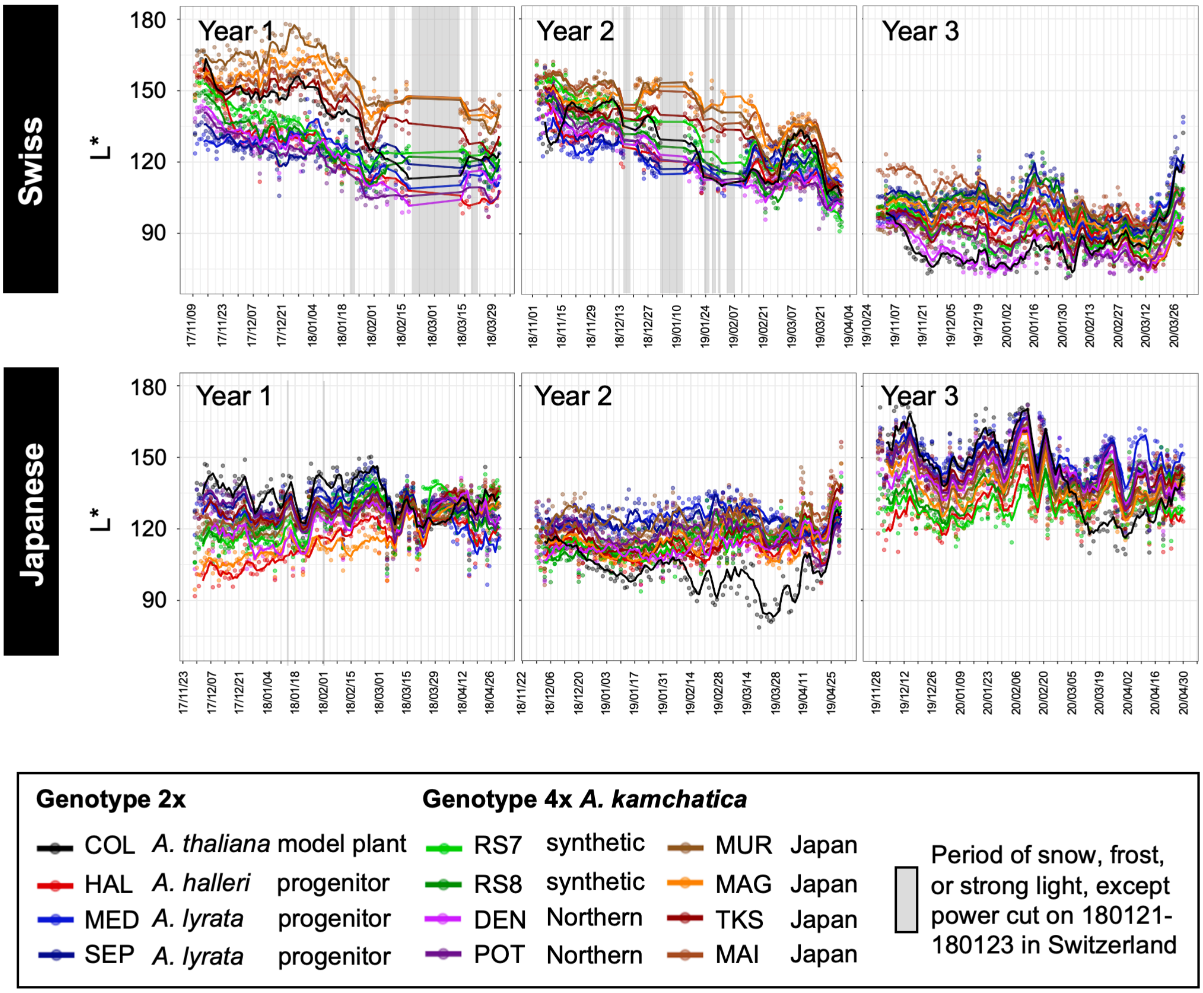

Supplementary Fig. 15. Time-series plots of the 5-day moving average of L* in plants of 12 *Arabidopsis* genotypes at the Swiss and Japanese sites in three seasons. The data points indicate the arithmetic mean. The number of plants is summarized in Supplementary Table 8. Source data are provided as a Source Data file.

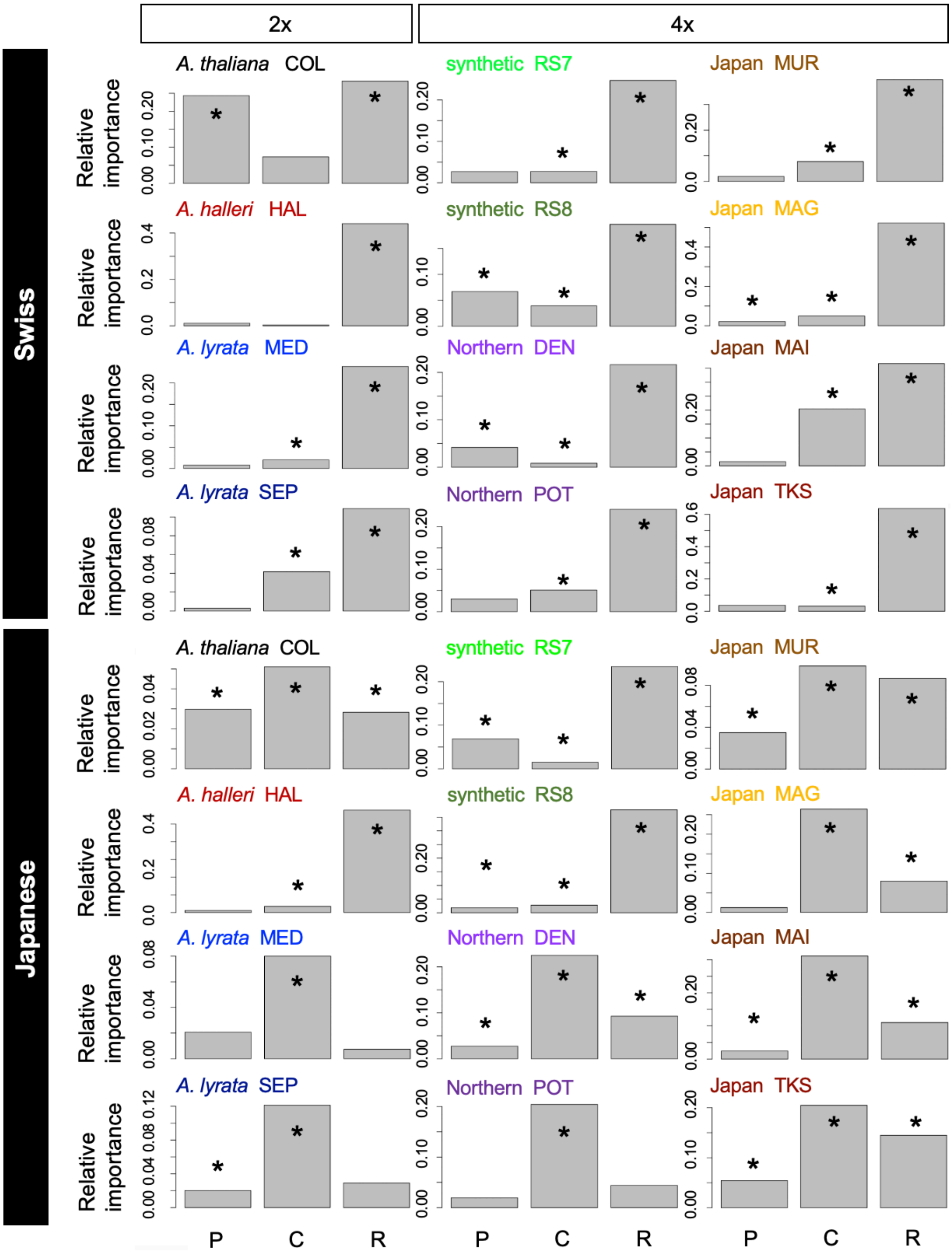

Supplementary Fig. 16. Environmental variables associated with the estimated anthocyanin content per area. The relative importance of the environmental variables on the estimated anthocyanin content for 12 genotypes of *Arabidopsis* at the Swiss and Japanese sites. P: Precipitation, C: Coldness, R: Radiation. The genotype code in three capital letters is indicated for each genotype. 2x indicates diploid and 4x indicates allotetraploid *A. kamchatica*. Significant variables based on confidence intervals calculated with bias-corrected and accelerated (BCa) bootstrapping are indicated with asterisks. *n* = 663 for the regression analysis of each genotype. Source data are provided as a Source Data file.

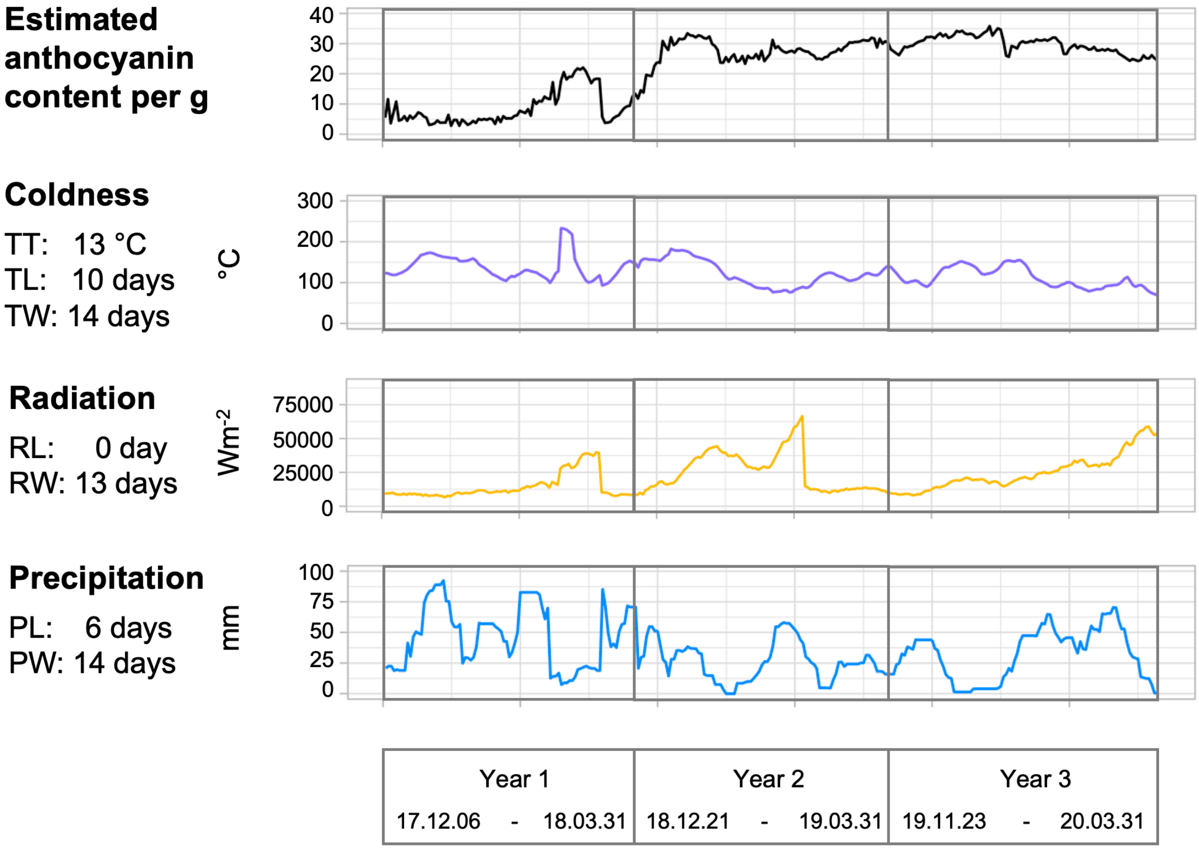

Supplementary Fig. 17. Time-series plots of the estimated relative anthocyanin content per weight and the environmental variables with best parameter combinations for the model plant *Arabidopsis thaliana* in three years in the Swiss site. TT: temperature threshold, TL: temperature lag, TW: temperature window, RL: radiation lag, RW: radiation window, PL: precipitation lag, PW: precipitation window. See Fig. 6a for the calculation of the environmental variables using the parameters. n = 663. Source data are provided as a Source Data file.

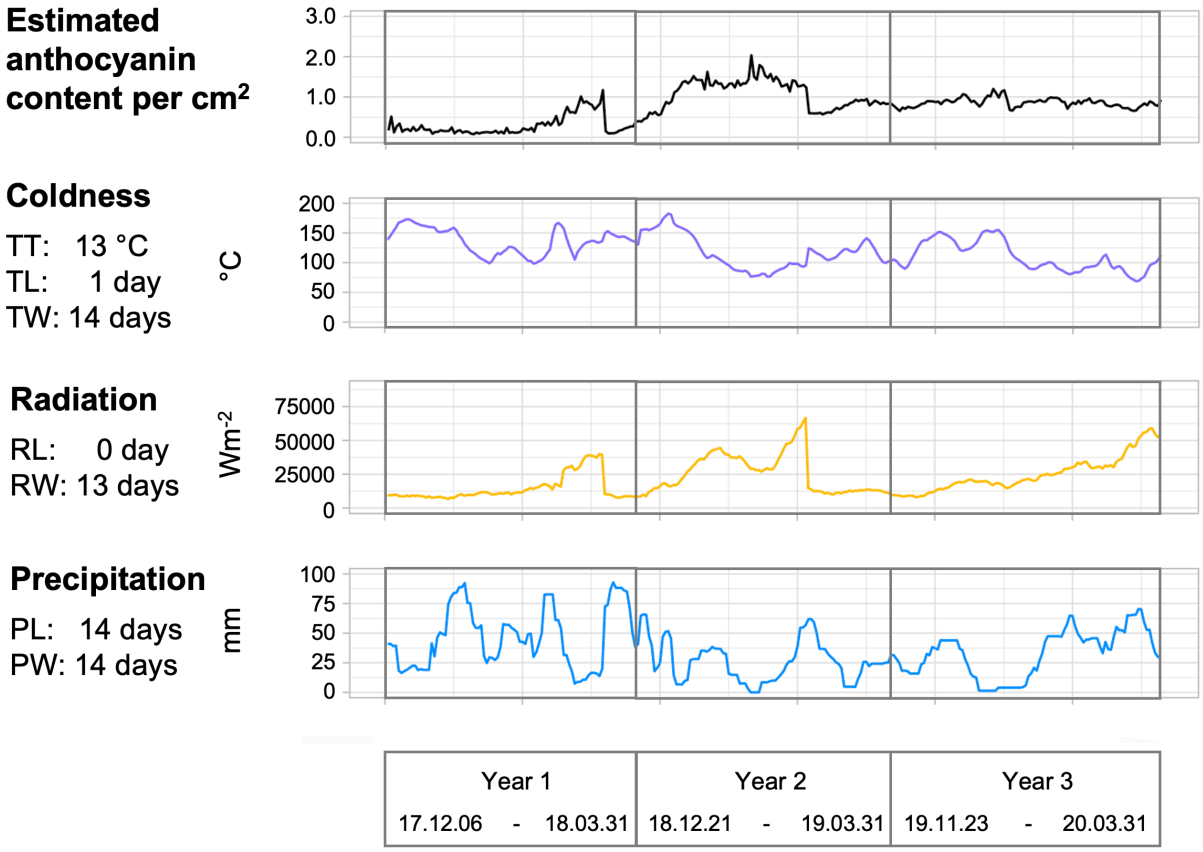

Supplementary Fig. 18. Time-series plots of the estimated anthocyanin content per area and the environmental variables with best parameter combinations for the model plant *Arabidopsis thaliana* in three years in the Swiss site. TT: temperature threshold, TL: temperature lag, TW: temperature window, RL: radiation lag, RW: radiation window, PL: precipitation lag, PW: precipitation window. See Fig. 6a for the calculation of the environmental variables using the parameters. n = 663. Source data are provided as a Source Data file.

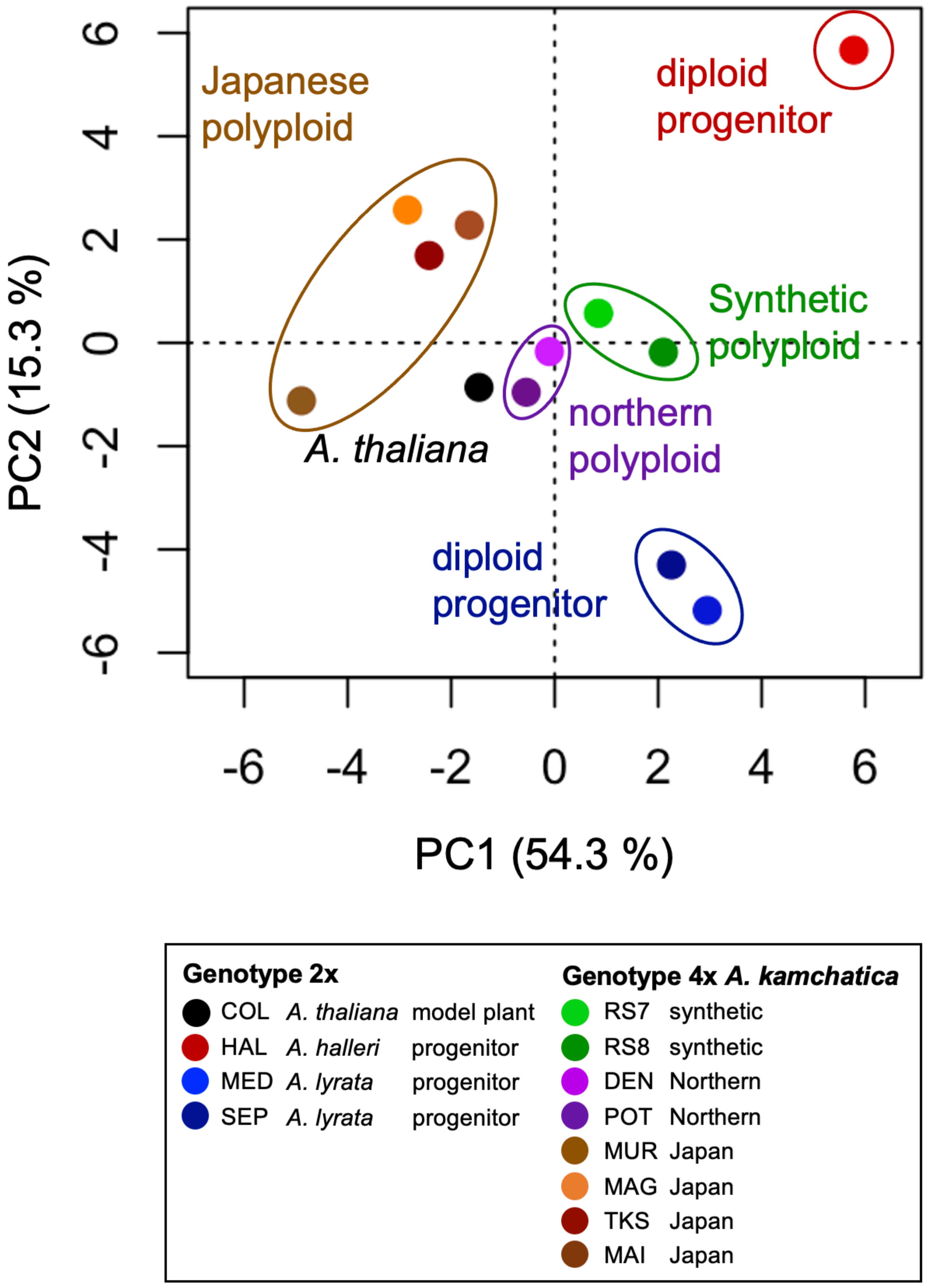

Supplementary Fig. 19. Principal component analysis plot of time-series estimated relative anthocyanin content per area of *Arabidopsis.* Genotype averages are shown. The data from the Swiss and Japanese sites in three seasons are merged. Source data are provided as a Source Data file.

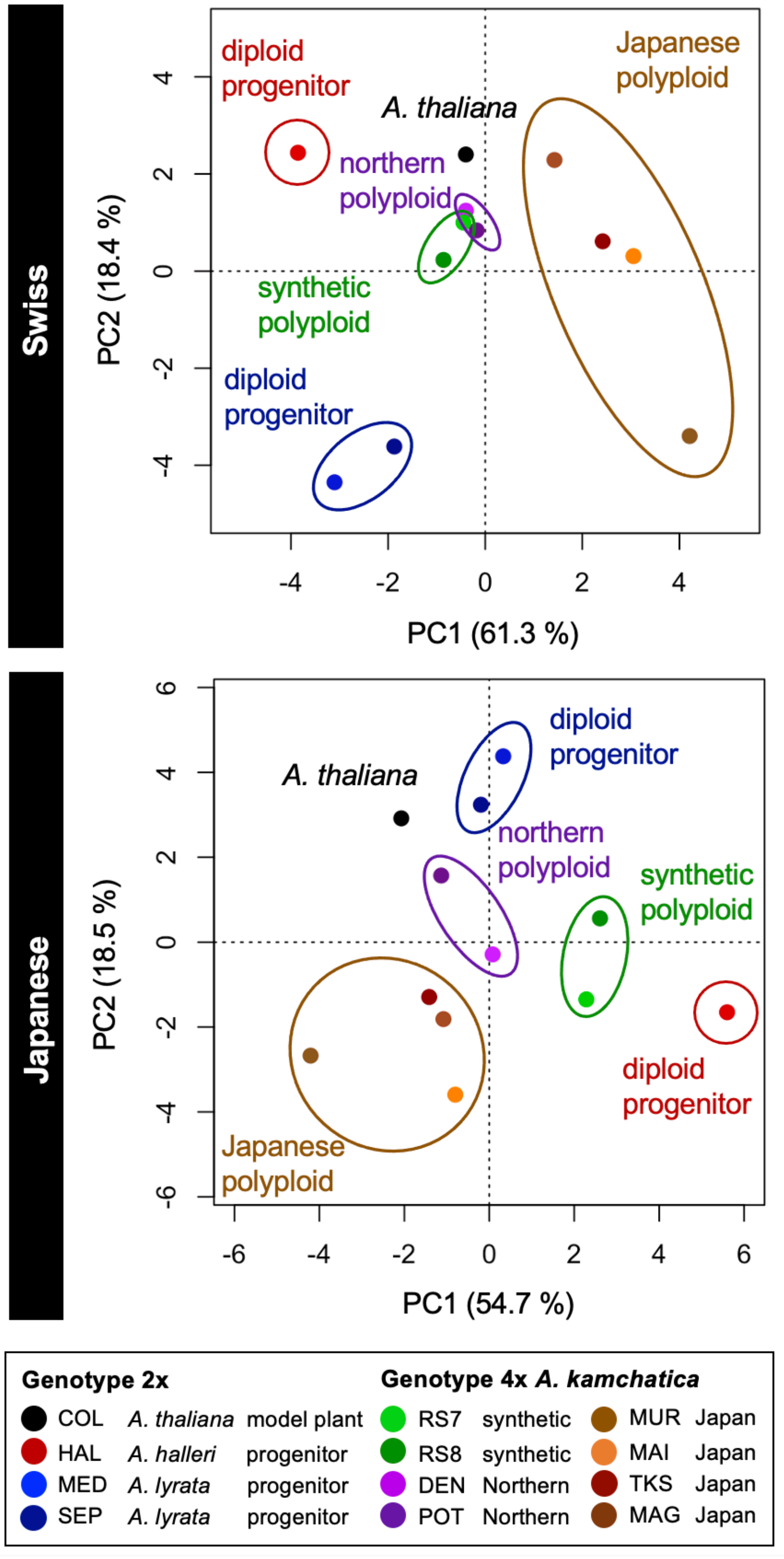

Supplementary Fig. 20. Principal component analysis plot of time-series estimated relative anthocyanin content per weight of *Arabidopsis*. Genotype averages are shown. The data from the three seasons are merged for Swiss and Japanese sites, respectively. Source data are provided as a Source Data file.

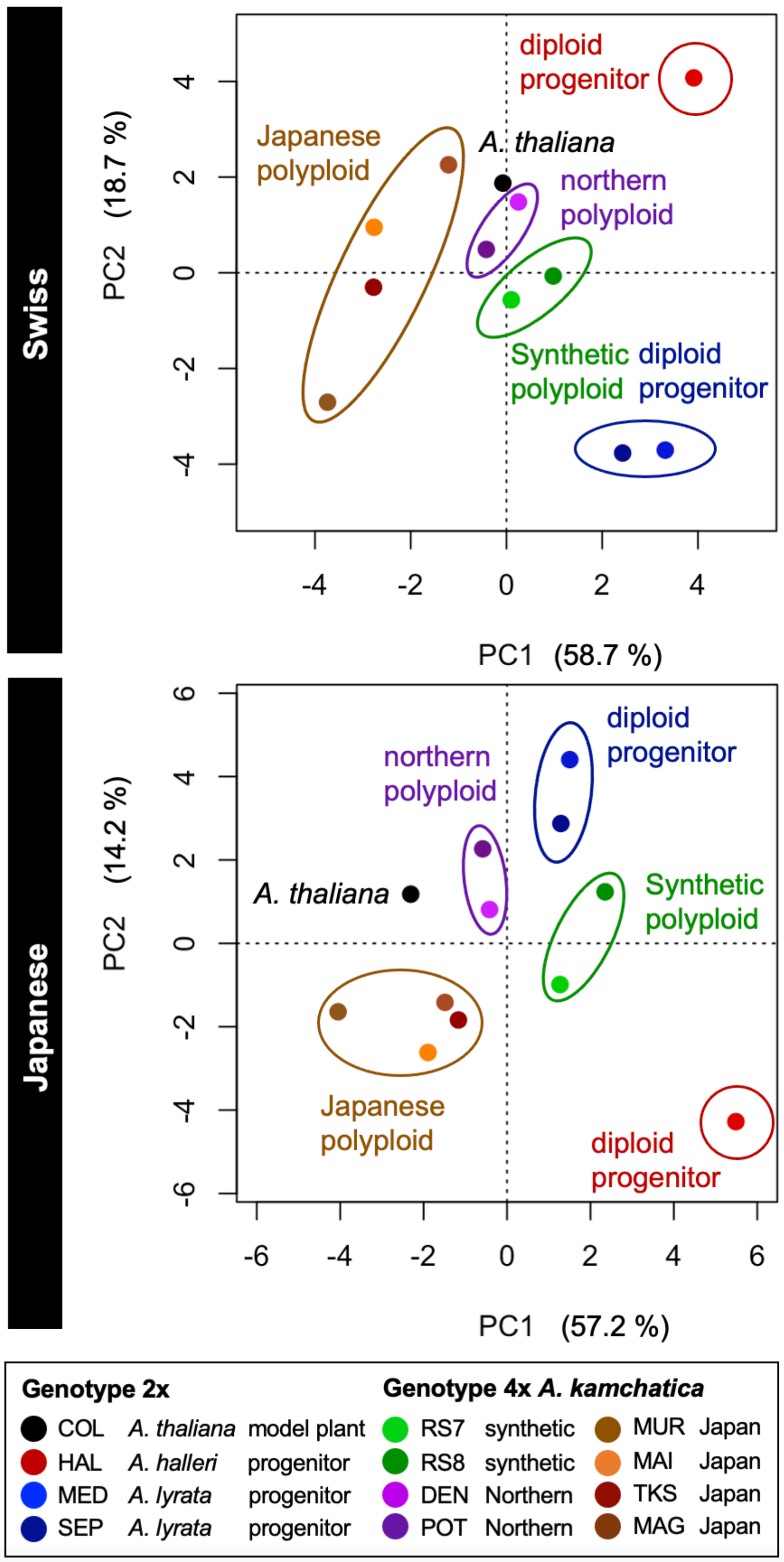

Supplementary Fig. 21. Principal component analysis plot of time-series estimated relative anthocyanin content per area of *Arabidopsis*. Genotype averages are shown. The data from the three seasons are merged for Swiss and Japanese sites, respectively. Source data are provided as a Source Data file.

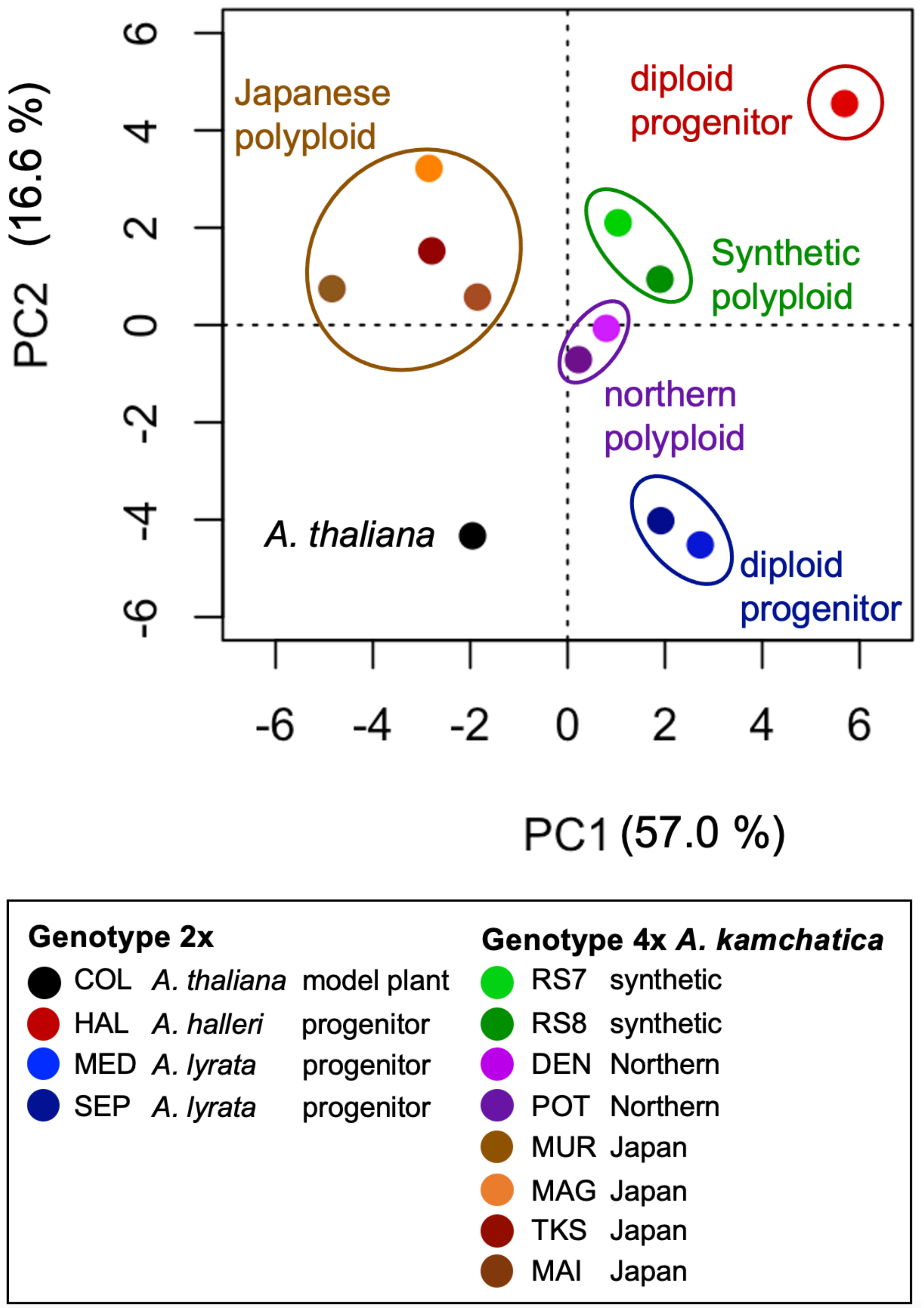

Supplementary Fig. 22. Principal component analysis plot of time-series a* of *Arabidopsis*. Genotype averages are shown. The data from the Swiss and Japanese sites in three seasons are merged. Source data are provided as a Source Data file.

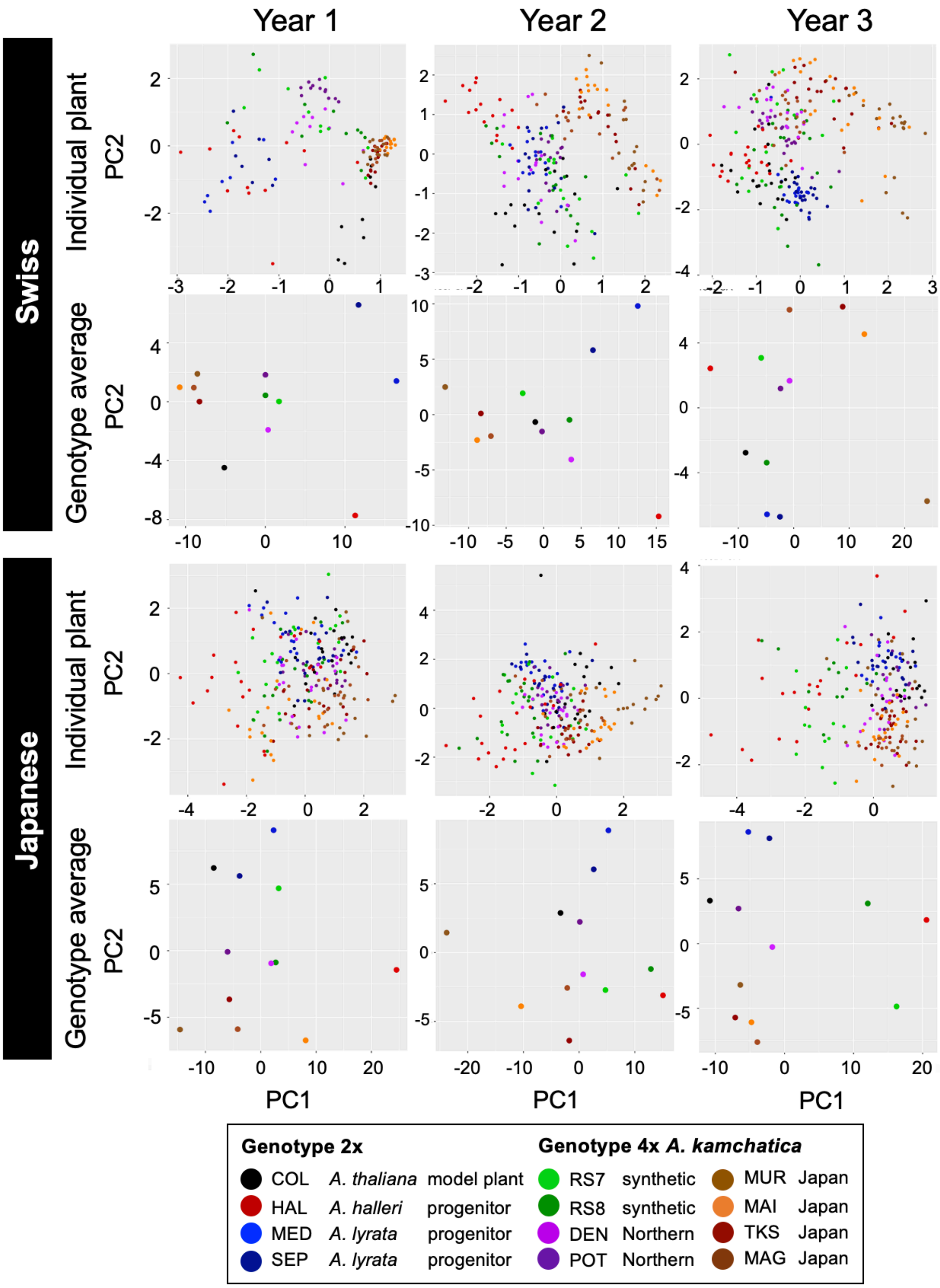

Supplementary Fig. 23. Principal component analysis (PCA) plots for estimated relative anthocyanin content per weight of 12 *Arabidopsis* genotypes at individual plant levels and of genotype average for Years 1, 2, and 3 at the Swiss and Japanese sites. Because new sets of plants were grown each year and therefore the individuals were not consistent across years or country, year and country are separately plotted. Missing values at individual-level data were imputed using Bayesian PCA. The proportion of missing values was 10.9%, 11.7%, and 12.9% for Switzerland Years 1, 2, and 3, and 5.0%, 6.0%, and 4.0% for Japan Years 1, 2, and 3, respectively. Source data are provided as a Source Data file.

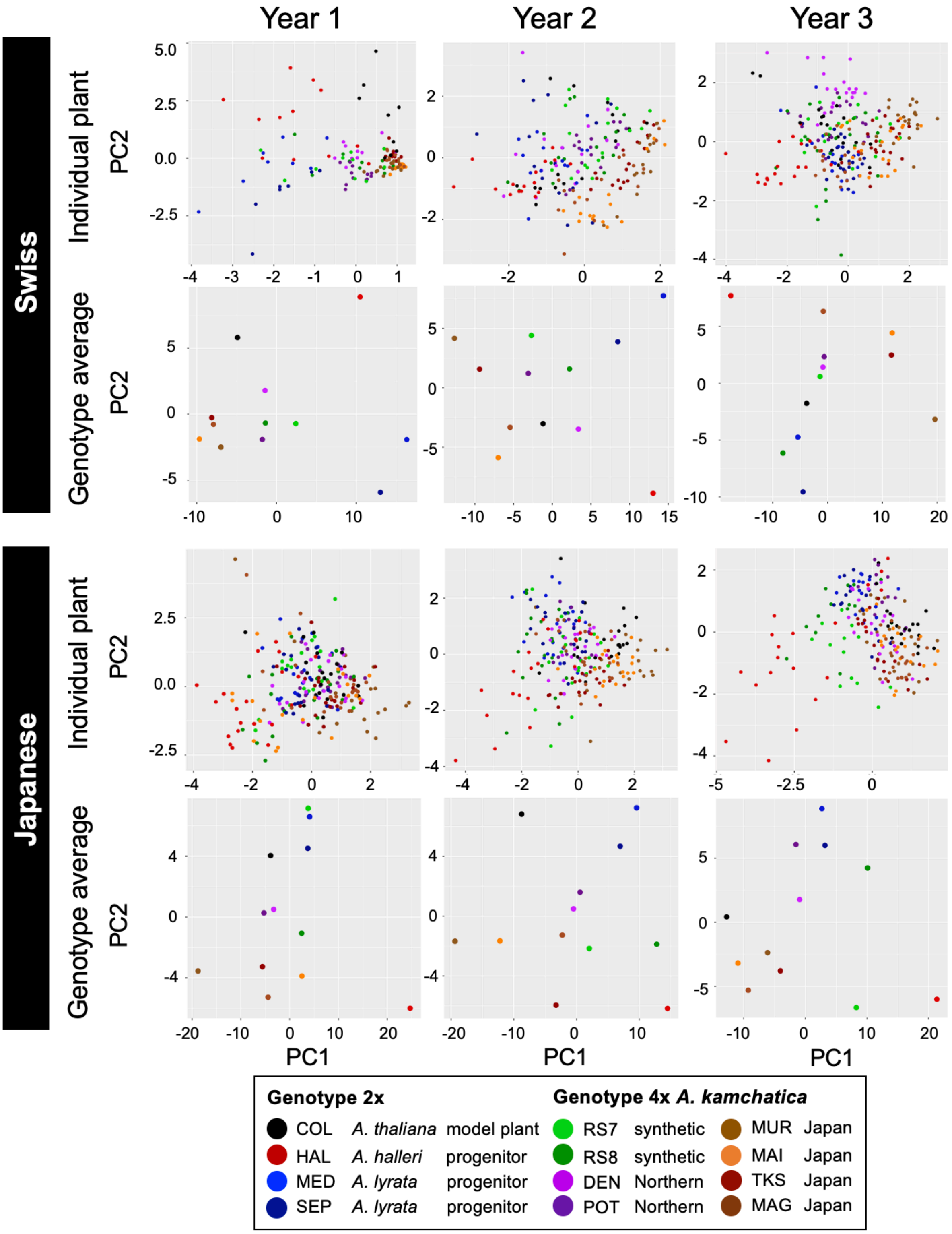

Supplementary Fig. 24. Principal component analysis (PCA) plots for estimated anthocyanin content per area of 12 *Arabidopsis* genotypes at individual plant levels and of genotype average for Years 1, 2, and 3 at the Swiss and Japanese sites. Because new sets of plants were grown each year and therefore the individuals were not consistent across years or country, year and country are separately plotted. Missing values at individual-level data were imputed using Bayesian PCA. The proportion of missing values was 10.9%, 11.7%, and 12.9% for Switzerland Years 1, 2, and 3, and 5.0%, 6.0%, and 4.0% for Japan Years 1, 2, and 3, respectively. Source data are provided as a Source Data file.

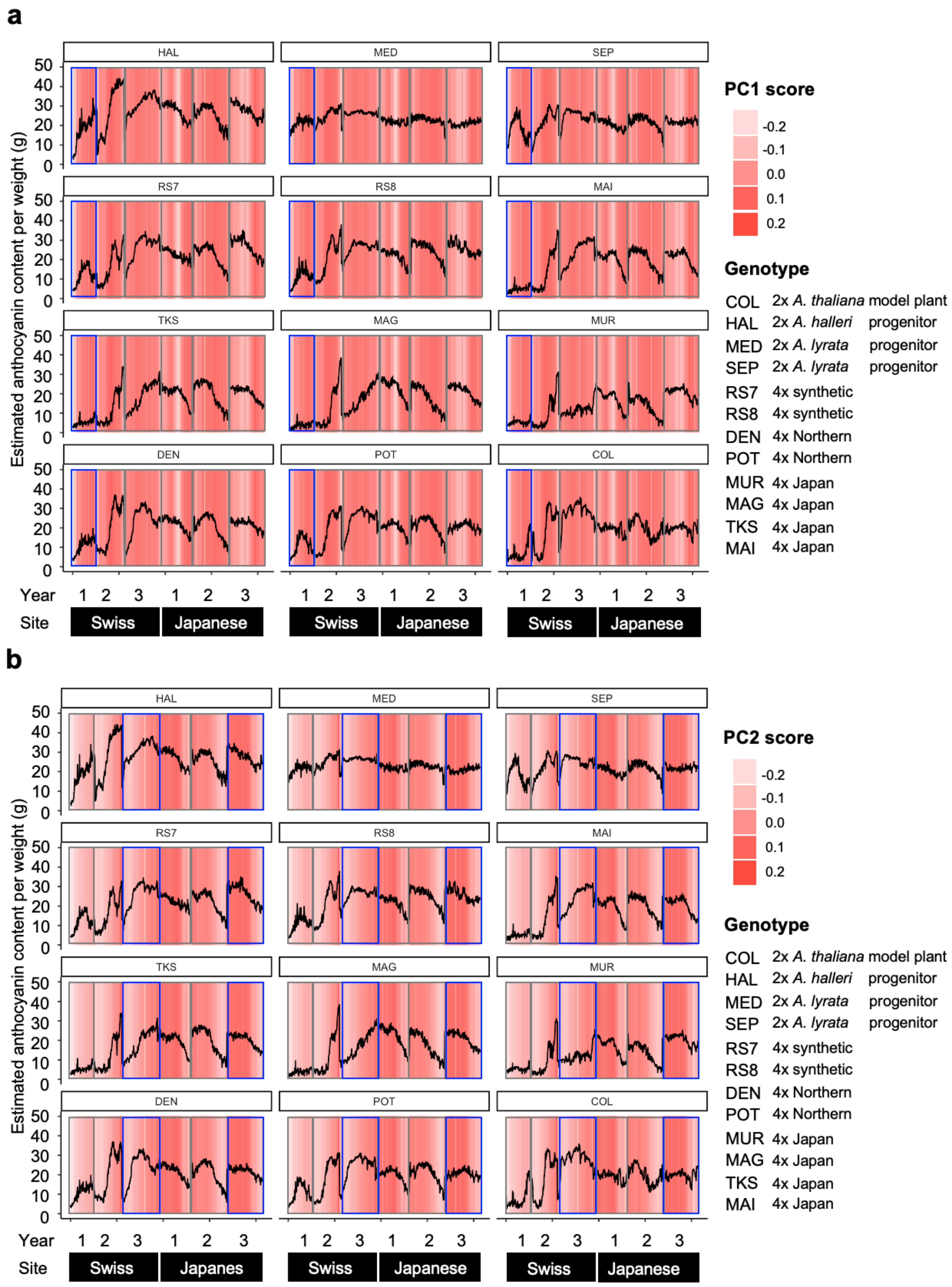

Supplementary Fig. 25. Time-series plots of the estimated relative anthocyanin content per weight for 12 genotypes of *Arabidopsis* overlaid on **(a)** Principal component (PC) 1 scores and **(b)** PC2 scores. 2x and 4x indicate diploid and allotetraploid, respectively. Blue rectangles indicate the period with distinct differences among genotypes and high PC scores. In addition, the PC2 score seemingly correlated with the estimated anthocyanin content throughout the years and sites. Source data are provided as a Source Data file.

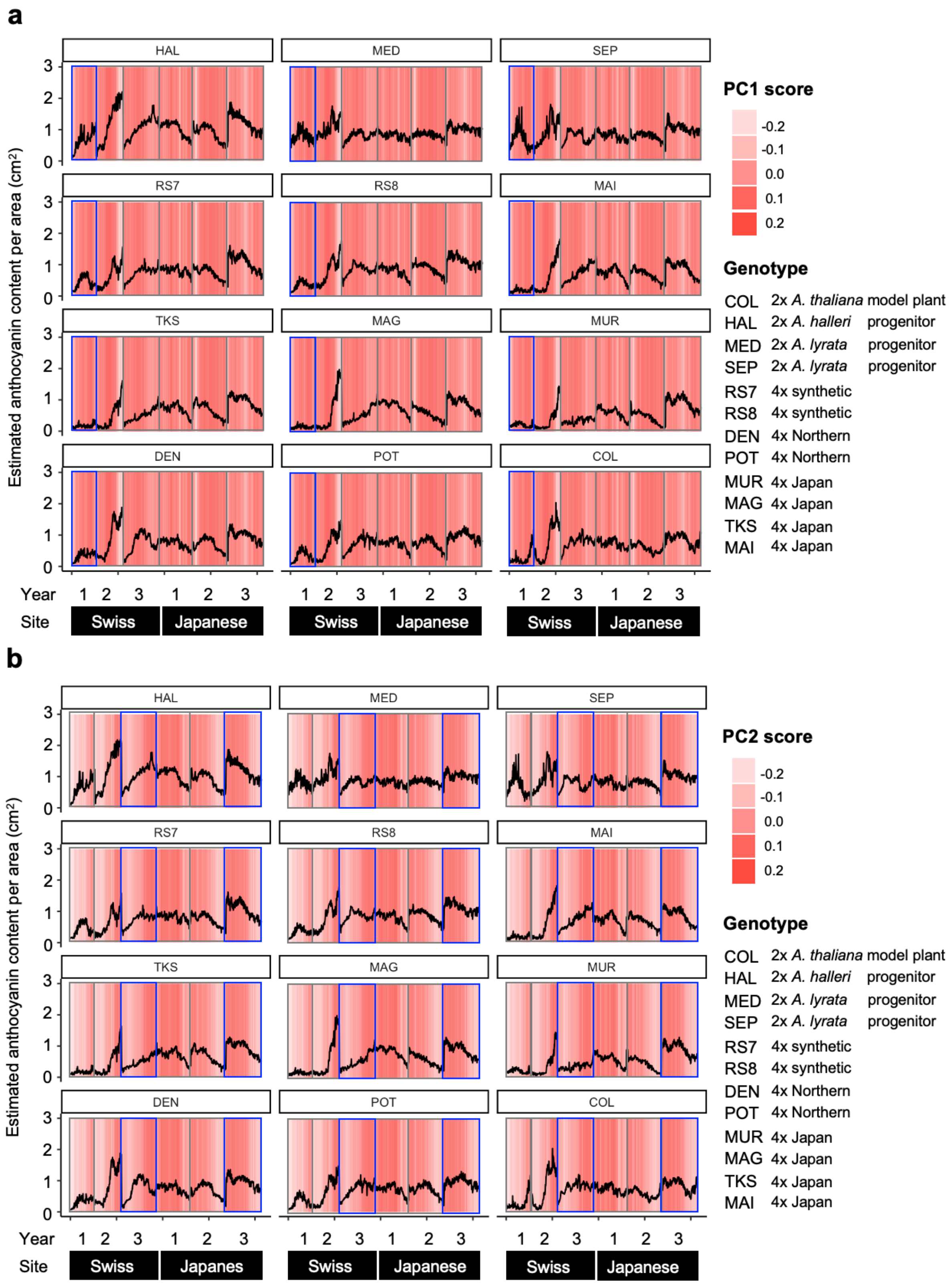

Supplementary Fig. 26. Time-series plots of the estimated anthocyanin content per area for 12 genotypes of *Arabidopsis* overlaid on **(a)** Principal component (PC) 1 scores and **(b)** PC2 scores. 2x and 4x indicate diploid and allotetraploid, respectively. Blue rectangles indicate the period with distinct differences among genotypes and high PC scores. In addition, the PC2 score seemingly correlated with the estimated anthocyanin content throughout the years and sites. Source data are provided as a Source Data file.

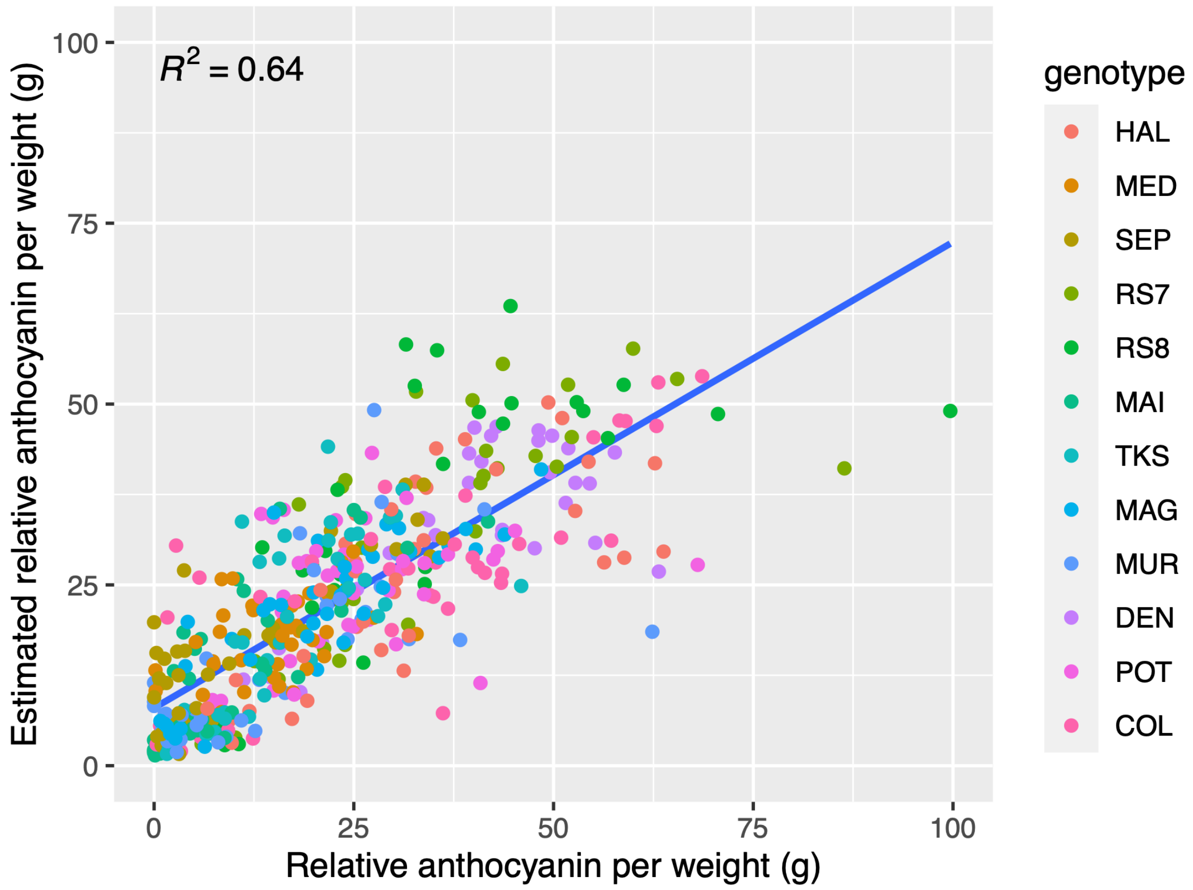

Supplementary Fig. 27. The result of the leave-one-out cross-validation of the random forest model to estimate the relative anthocyanin content per weight of 12 *Arabidopsis* genotypes. The scatter plot shows the relation between the relative anthocyanin per weight and the estimated relative anthocyanin per weight when one data point is excluded per time using the random forest model. R-squared (R^2) is indicated in the figure. $n = 451$. Source data are provided as a Source Data file.

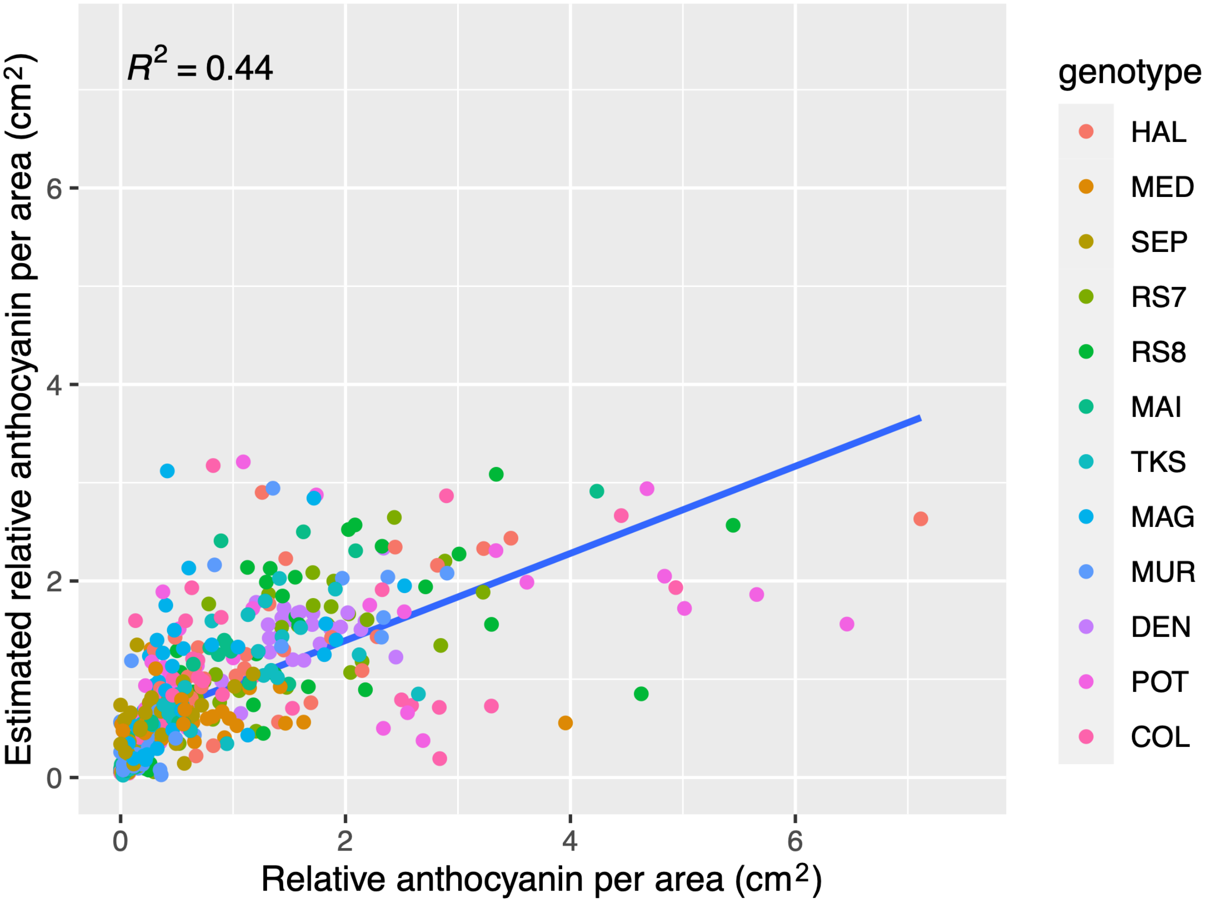

Supplementary Fig. 28. The result of the leave-one-out cross-validation of the random forest model to estimate the relative anthocyanin per area of 12 *Arabidopsis* genotypes. The scatter plot shows the relation between the relative anthocyanin per mm2 and the estimated relative anthocyanin per area when one data point is excluded per time using the random forest model. R-squared (R^2) is indicated in the figure. n = 451. Source data are provided as a Source Data file.

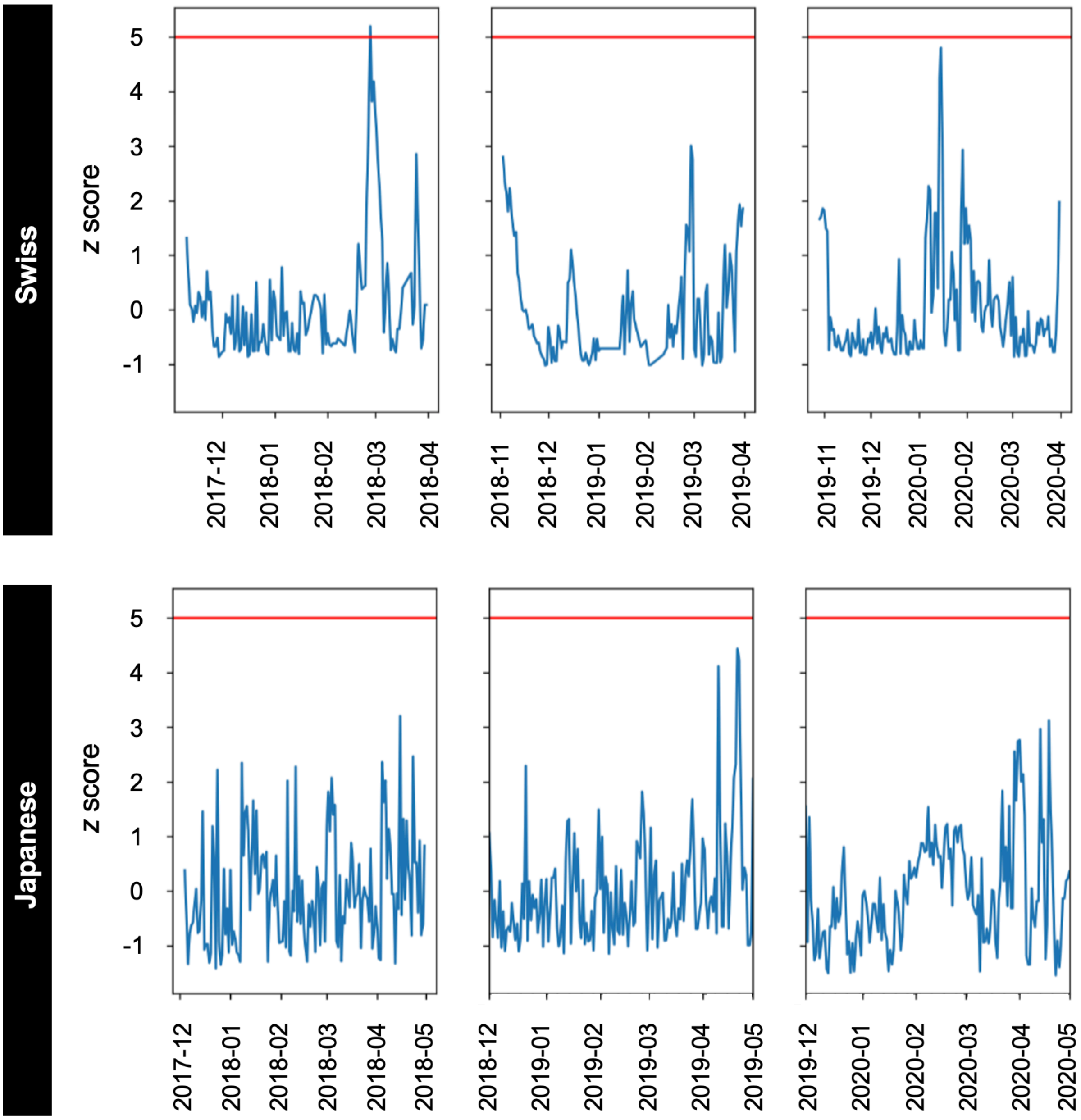

Supplementary Fig. 29. Detection of the period of drastic value changes (outliers) in the time-series plots for plant area of 12 *Arabidopsis* genotypes at the Swiss and Japanese sites in three seasons using nearest neighbor imputation. Red lines indicate the threshold value of $z = 5$. Source data are provided as a Source Data file.

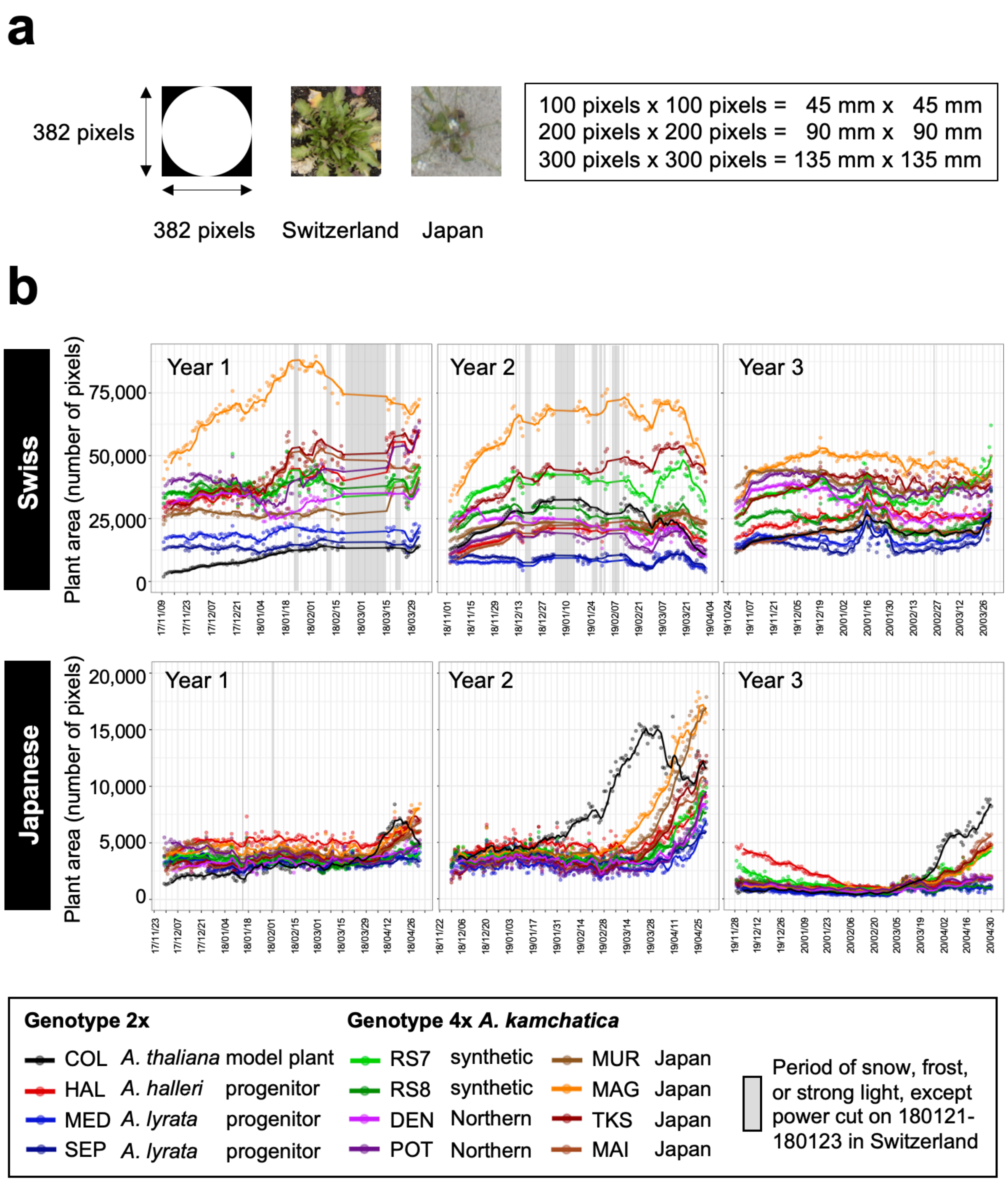

Supplementary Fig. 30. The plant area of 12 *Arabidopsis* genotypes at the Swiss and Japanese sites. **(a)** The details of the measurement in pixels with representative images and the conversion to the area in mm^2. **(b)** Time-series plots of the 5-day moving average of the plant area of 12 genotypes at the Swiss and Japanese sites in three seasons. Note the Y-axis scale difference between sites. The data points indicate the arithmetic mean. The number of plants is summarized in Supplementary Table 8. Source data are provided as a Source Data file.

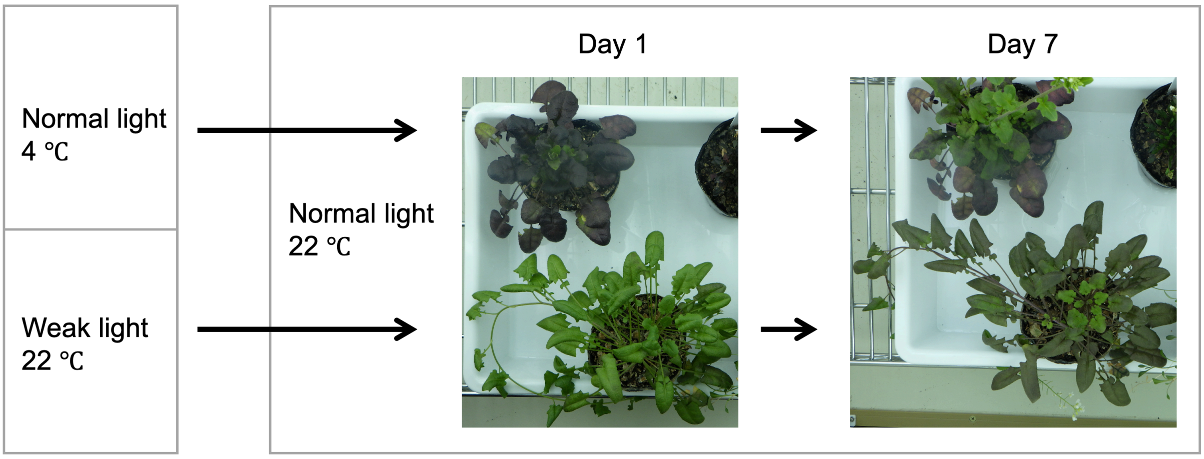

Supplementary Fig. 31. Images of individuals of *Arabidopsis halleri* W302 subject to experiments in which temperature and light intensity were manipulated. The plant above was first kept at a low temperature and then brought to a benign temperature under a normal light condition (ca. 100 μmol m^{-2} s^{-1}) to examine the effect of temperature on plant color. The plant below was first kept at weak light (ca. 40 μmol m^{-2} s^{-1}) and then brought to normal light under benign temperature to examine the effect of light intensity on plant color. The day length was kept at a short-day condition throughout the experiment with 10h:14h = light: dark.

Supplementary Table 1. The name of the species, ploidy level, genotype code, altitude of origin, latitude of origin, and longitude of origin of the genotypes of *Arabidopsis* used in the study. RS7 and RS8 were synthesized in the laboratory and thus have no information on the altitude, latitude, or longitude of origin. We used the lab accession Col-0 for *A. thaliana*.

Species	Ploidy	Country	Code	Altitude (m)	Latitude	Longitude
A. halleri subsp. *gemmifera*	2x	Japan	HAL	140	34°53' N	135°21' E
A. lyrata subsp. *petraea*	2x	Russia	MED	40	68°48' N	160°18' E
A. lyrata subsp. *petraea*	2x	Russia	SEP	14	70°43' N	148°45' E
A. kamchatica	4x	synthetic	RS7	-	-	-
A. kamchatica	4x	synthetic	RS8	-	-	-
A. kamchatica subsp. *kamchatica*	4x	Russia	DEN	265	54°15' N	158°07' E
A. kamchatica subsp. *kamchatica*	4x	U.S.	POT	5	61°03' N	149°48' W
A. kamchatica subsp. *kamchatica*	4x	Japan	MUR	2421	36°34' N	137°35' E
A. kamchatica subsp. *kamchatica*	4x	Japan	MAG	543	35°15' N	138°19' E
A. kamchatica subsp. *kawasakiana*	4x	Japan	MAI	87	35°08' N	135°59' E
A. kamchatica subsp. *kawasakiana*	4x	Japan	TKS	85	35°27' N	136°03' E
A. thaliana	2x	-	COL	-	-	-

Supplementary Table 2. The summary of the Dice coefficient of the outcome of segmentation using different DNNs on 4,100 images of soil background from the Swiss site.

Category	U-Net	Resnet101 + U-Net	EfficientNet-B7 + U-Net	SINet	DANet
average	0.662	0.778	0.808	0.673	0.819
minimum	0.186	0.513	0.544	0.294	0.562
maximum	0.919	0.952	0.952	0.889	0.962

Supplementary Table 3. The summary of assessment indices of the outcome of segmentation using DANet on 7,500 images from the Swiss (soil and sand backgrounds) and Japanese (sand and humus background) sites.

Category	Dice	Sensitivity	Specificity
mean	0.5767	0.8655	0.9544
standard deviation	0.2441	0.1292	0.0190
maximum	0.9098	0.9912	0.9804
minimum	0.1077	0.4760	0.9150

Supplementary Table 4. The environmental parameters of the best regression model for estimated anthocyanin content per weight and the effect of environmental factors in the model (*n* = 663 for each genotype). 2x and 4x indicate diploid and allotetraploid, respectively. Code indicates the genotype code. PL: precipitation lag, PW: precipitation window, TT: temperature threshold, TL: temperature lag, TW: temperature window, RL: radiation lag, RW: radiation window, P: precipitation, C: coldness (calculated from TT, TL, and TW), R: radiation, The unit is the number of days for all parameters. *: significant effect based on confidence intervals calculated with bias-corrected and accelerated (BCa) bootstrapping.

				Best environmental parameter							Environmental factor effect		
Site	Description	Species	Code	PL	PW	TT	TL	TW	RL	RW	P	C	R
Swiss	model plant 2x	*A. thaliana*	COL	6	14	13	10	14	0	13	*	*	*
Swiss	progenitor 2x	*A. halleri*	HAL	0	14	13	4	14	0	14	*	*	*
Swiss	progenitor 2x	*A. lyrata*	MED	0	4	1	14	14	0	3	*	*	*
Swiss	progenitor 2x	*A. lyrata*	SEP	0	1	1	14	14	0	1	*	*	*
Swiss	synthetic 4x	*A. kamchatica*	RS7	0	2	1	14	14	0	2	*	*	*
Swiss	synthetic 4x	*A. kamchatica*	RS8	0	4	1	14	14	0	4	*	*	*
Swiss	Northern 4x	*A. kamchatica*	DEN	0	4	13	0	3	0	4	*	*	*
Swiss	Northern 4x	*A. kamchatica*	POT	0	4	1	14	14	0	4	*	*	*
Swiss	Japanese 4x	*A. kamchatica*	MUR	0	1	10	10	14	0	2	*	*	*
Swiss	Japanese 4x	*A. kamchatica*	MAG	0	1	13	3	14	0	6	N.S.	*	*
Swiss	Japanese 4x	*A. kamchatica*	MAI	0	1	13	2	14	0	2	N.S.	*	*
Swiss	Japanese 4x	*A. kamchatica*	TKS	3	3	13	2	14	0	6	N.S.	*	*
Japanese	model plant 2x	*A. thaliana*	COL	2	2	10	1	4	0	1	*	*	N.S.
Japanese	progenitor 2x	*A. halleri*	HAL	8	8	13	0	12	0	14	N.S.	*	*
Japanese	progenitor 2x	*A. lyrata*	MED	0	1	13	0	11	0	2	N.S.	*	N.S.
Japanese	progenitor 2x	*A. lyrata*	SEP	0	2	13	0	7	1	1	N.S.	*	N.S.
Japanese	synthetic 4x	*A. kamchatica*	RS7	9	7	4	0	9	14	14	*	*	*
Japanese	synthetic 4x	*A. kamchatica*	RS8	14	12	4	0	5	14	14	*	*	*
Japanese	Northern 4x	*A. kamchatica*	DEN	0	14	13	4	14	0	14	N.S.	*	*
Japanese	Northern 4x	*A. kamchatica*	POT	1	1	13	1	14	1	1	N.S.	*	N.S.
Japanese	Japanese 4x	*A. kamchatica*	MUR	0	2	13	0	7	0	2	*	*	*
Japanese	Japanese 4x	*A. kamchatica*	MAG	0	2	13	0	14	0	2	N.S.	*	*
Japanese	Japanese 4x	*A. kamchatica*	MAI	0	2	13	1	14	0	2	*	*	*
Japanese	Japanese 4x	*A. kamchatica*	TKS	0	8	13	1	14	0	8	*	*	*

Supplementary Table 5. The environmental parameters of the best regression model for estimated anthocyanin content per area and the effect of environmental factors in the model (*n* = 663 for each genotype). 2x and 4x indicate diploid and allotetraploid, respectively. PL: precipitation lag, PW: precipitation window, TT: temperature threshold, TL: temperature lag, TW: temperature window, RL: radiation lag, RW: radiation window, P: precipitation, C: coldness (calculated from TT, TL, and TW), R: radiation, The unit is the number of days for all parameters. *: significant effect based on confidence intervals calculated with bias-corrected and accelerated (BCa) bootstrapping.

				Best environmental parameter							Environmental factor effect		
Site	Description	Genotype	Code	PL	PW	TT	TL	TW	RL	RW	P	C	R
Swiss	model plant 2x	*A. thaliana*	COL	14	14	13	1	14	0	13	*	N.S.	*
Swiss	progenitor 2x	*A. halleri*	HAL	0	2	1	14	14	0	4	N.S.	N.S.	*
Swiss	progenitor 2x	*A. lyrata*	MED	1	1	13	1	14	0	1	N.S.	*	*
Swiss	progenitor 2x	*A. lyrata*	SEP	1	1	13	1	14	0	1	N.S.	*	*
Swiss	synthetic 4x	*A. kamchatica*	RS7	0	1	1	14	14	0	2	N.S.	*	*
Swiss	synthetic 4x	*A. kamchatica*	RS8	0	4	1	14	14	0	4	*	*	*
Swiss	Northern 4x	*A. kamchatica*	DEN	0	4	1	7	4	0	4	*	*	*
Swiss	Northern 4x	*A. kamchatica*	POT	0	1	1	14	14	0	2	N.S.	*	*
Swiss	Japan 4x	*A. kamchatica*	MUR	0	2	7	14	14	0	2	N.S.	*	*
Swiss	Japan 4x	*A. kamchatica*	MAG	0	6	1	14	14	0	6	*	*	*
Swiss	Japan 4x	*A. kamchatica*	MAI	1	3	13	2	14	0	4	N.S.	*	*
Swiss	Japan 4x	*A. kamchatica*	TKS	0	5	1	14	14	0	6	N.S.	*	*
Japanese	model plant 2x	*A. thaliana*	COL	2	2	1	0	2	0	4	*	*	*
Japanese	progenitor 2x	*A. halleri*	HAL	1	1	1	12	13	0	14	N.S.	*	*
Japanese	progenitor 2x	*A. lyrata*	MED	1	1	13	0	5	0	2	N.S.	*	N.S.
Japanese	progenitor 2x	*A. lyrata*	SEP	1	1	13	0	5	0	2	*	*	N.S.
Japanese	synthetic 4x	*A. kamchatica*	RS7	0	4	4	5	5	0	4	*	*	*
Japanese	synthetic 4x	*A. kamchatica*	RS8	1	1	4	0	14	0	14	*	*	*
Japanese	Northern 4x	*A. kamchatica*	DEN	1	1	13	0	8	0	2	*	*	*
Japanese	Northern 4x	*A. kamchatica*	POT	1	1	13	0	8	0	2	N.S.	*	N.S.
Japanese	Japan 4x	*A. kamchatica*	MUR	0	2	13	0	4	0	2	*	*	*
Japanese	Japan 4x	*A. kamchatica*	MAG	1	1	13	0	8	0	2	N.S.	*	*
Japanese	Japan 4x	*A. kamchatica*	MAI	1	1	13	0	14	0	2	*	*	*
Japanese	Japan 4x	*A. kamchatica*	TKS	0	2	13	0	5	0	2	*	*	*

Supplementary Table 6. The settings of the camera (RICOH WG-40) used for image acquisition. The Interval was set at either 90min 0sec, or 60min 0sec, depending on the routine at each site.

Mode	Category	Subcategory	Setting
General shooting	General shooting mode		Interval shot
	Setting of the Interval Shot	Interval	90min 0sec
		Number of shots	1000
		Start Delay	Circumstance dependent
	Flash mode		Flash OFF
Recording	Image Tone		Natural
	Recorded Pixel		16M
	Quality Level		★★★
	White Balance		AWB
	AF Setting	Focusing Area	whole
		Auto Macro	ON
		Focus Assist	OFF
	AE Metering		Multi-segment metering
	Sensitivity		AUTO
	AUTO ISO Range		ISO125-1600
	EV Compensation		±0.0
	D-Range Setting	Highlight	Auto
		Shadow	Auto
	Pixel Track SR		ON
	Face Detection		OFF
	Blink Detection		OFF
	Digital Zoom		OFF
	Instant Review		OFF
	Memory	Make all items/settings	ON
	Green Button		ON
	Sharpness		0
	Saturation		0
	Contrast		0
	Date Imprint		OFF
	IQ Enhance		OFF
	Macro Light		OFF

Supplementary Table 7. The details of the R packages used for data visualization and analyses.

R package	Version	Reference
randomForest	4.6.12	Liaw A, Wiener M (2002). "Classification and Regression by randomForest." R News, 2(3), 18-22. https://CRAN.R-project.org/doc/Rnews
zoo	1.8.9	Zeileis A, Grothendieck G (2005). "zoo: S3 Infrastructure for Regular and Irregular Time Series." Journal of Statistical Software, 14(6), 1–27. doi: 10.18637/jss.v014.i06
dplyr	1.0.8	Wickham H, François R, Henry L, Müller K (2022). dplyr: A Grammar of Data Manipulation. https://dplyr.tidyverse.org, https://github.com/tidyverse/dplyr
ggplot2	3.3.5	Wickham H (2016). ggplot2: Elegant Graphics for Data Analysis. Springer-Verlag New York. ISBN 978-3-319-24277-4, https://ggplot2.tidyverse.org
tidyverse	1.3.1	Wickham H, Averick M, Bryan J, Chang W, McGowan LD, François R, Grolemund G, Hayes A, Henry L, Hester J, Kuhn M, Pedersen TL, Miller E, Bache SM, Müller K, Ooms J, Robinson D, Seidel DP, Spinu V, Takahashi K, Vaughan D, Wilke C, Woo K, Yutani H (2019). "Welcome to the tidyverse." Journal of Open Source Software, 4(43), 1686. doi:10.21105/joss.01686
pcaMethods	1.86.0	Stacklies W, Redestig H, Scholz M, Walther D, Selbig J (2007). "pcaMethods – a Bioconductor package providing PCA methods for incomplete data." Bioinformatics, 23, 1164–1167.
imputeTS	3.2	Moritz S, Bartz-Beielstein T (2017). "imputeTS: Time Series Missing Value Imputation in R." The R Journal, 9(1), 207–218. doi: 10.32614/RJ-2017-009
boot	1.3.28	Canty A, Ripley BD (2021). boot: Bootstrap R (S-Plus) Functions. R package version 1.3-28.<br>Davison AC, Hinkley DV (1997). Bootstrap Methods and Their Applications. Cambridge University Press, Cambridge. ISBN 0-521-57391-2, http://statwww.epfl.ch/davison/BMA/
relaimpo	2.2.6	Grömping U (2006). "Relative Importance for Linear Regression in R: The Package relaimpo." Journal of Statistical Software, 17(1), 1–27
reshape2	1.4.4	Wickham H (2007). "Reshaping Data with the reshape Package." Journal of Statistical Software, 21(12), 1–20. http://www.jstatsoft.org/v21/i12/

Supplementary Table 8. The name of the species, genotype code, and number of plants per site (Switzerland or Japan) per season (yr1, yr2, or yr3) of the genotypes of *Arabidopsis* used for time-series plots and the downstream analyses in the study.

		Switzerland			Japan		
Species	Code	yr1	yr2	yr3	yr1	yr2	yr3
A. halleri	HAL	12	16	20	20	20	20
A. lyrata	MED	8	14	21	20	20	16
A. lyrata	SEP	7	15	19	19	20	17
A. kamchatica	RS7	10	16	19	20	20	13
A. kamchatica	RS8	10	16	20	20	20	14
A. kamchatica	DEN	10	16	19	20	20	20
A. kamchatica	POT	12	16	20	20	18	14
A. kamchatica	MUR	12	16	20	20	19	20
A. kamchatica	MAG	12	16	20	20	20	19
A. kamchatica	MAI	12	16	20	20	20	18
A. kamchatica	TKS	12	16	20	20	20	20
A. thaliana	COL	9	16	20	20	20	19

Supplementary Table 9. The summary of devices used for collecting environmental data at the Japanese site.

Environmental factor	Product	Manufacturer	Distributor	Distributor location
air temperature	HMP 155A	Campbell Scientific INC.	TAIYO KEIKI Co., Ltd.	Saitama
radiation	CMP-3	PREDE Co., Ltd.	PREDE Co., Ltd.	Tokyo
precipitation	RT-5E	IKEDA KEIKI Co., Ltd.	IKEDA KEIKI Co., Ltd.	Tokyo
