# Supplementary material for: Seasonal pigment fluctuation in diploid and polyploid Arabidopsis revealed by machine learning-based phenotyping method PlantServation
Source: Nat Commun. 2023 Sep 22;14:5792. doi: 10.1038/s41467-023-41260-3 (PMC10517152; doi:10.1038/s41467-023-41260-3)
Supplement: Supplementary file 2 — Reporting Summary [file 41467_2023_41260_MOESM2_ESM.pdf]

nature portfolio

Corresponding author(s): Kentaro K. Shimizu, Rie Shimizu-Inatsugi

Last updated by author(s): 31 JUL 2023

Reporting Summary

Nature Portfolio wishes to improve the reproducibility of the work that we publish. This form provides structure for consistency and transparency in reporting. For further information on Nature Portfolio policies, see our Editorial Policies and the Editorial Policy Checklist.

Statistics

For all statistical analyses, confirm that the following items are present in the figure legend, table legend, main text, or Methods section.

n/a	Confirmed	
☐	☒	The exact sample size (n) for each experimental group/condition, given as a discrete number and unit of measurement
☐	☒	A statement on whether measurements were taken from distinct samples or whether the same sample was measured repeatedly
☐	☒	The statistical test(s) used AND whether they are one- or two-sided<br>*Only common tests should be described solely by name; describe more complex techniques in the Methods section.*
☐	☒	A description of all covariates tested
☐	☒	A description of any assumptions or corrections, such as tests of normality and adjustment for multiple comparisons
☐	☒	A full description of the statistical parameters including central tendency (e.g. means) or other basic estimates (e.g. regression coefficient) AND variation (e.g. standard deviation) or associated estimates of uncertainty (e.g. confidence intervals)
☐	☒	For null hypothesis testing, the test statistic (e.g. F, t, r) with confidence intervals, effect sizes, degrees of freedom and P value noted<br>*Give P values as exact values whenever suitable.*
☒	☐	For Bayesian analysis, information on the choice of priors and Markov chain Monte Carlo settings
☒	☐	For hierarchical and complex designs, identification of the appropriate level for tests and full reporting of outcomes
☐	☒	Estimates of effect sizes (e.g. Cohen's d, Pearson's r), indicating how they were calculated

Our web collection on statistics for biologists contains articles on many of the points above.

Software and code

Policy information about availability of computer code

Data collection	We used open-source software as well as custom-made codes to process and analyze the data collected in this study. The software versions and the details of the custom codes are described in the manuscript.
Data analysis	We used Python version 3.6.6, 3.8.3, and 3.9.10, R version 4.1.0 and 4.2.0, and Fiji (Image J) ver. 1.52n.<br><br>The scripts used for the analyses and figures of non-image data are provided as Supplementary Code 1. The PlantServation demo set (ca. 600 MB) including scripts and demo data for PlantServation software is available at Zenodo accessible via Dryad repository [DOI: 10.5061/dryad.h70rxwdnk]. Note that visiting the Dryad link automatically starts the downloading process of ca. 250 GB Dryad dataset, but this process can be canceled and only the PlantServation demo set can be downloaded by clicking the PlantServation_demo_set.zip on the website.

For manuscripts utilizing custom algorithms or software that are central to the research but not yet described in published literature, software must be made available to editors and reviewers. We strongly encourage code deposition in a community repository (e.g. GitHub). See the Nature Portfolio guidelines for submitting code & software for further information.

Data

Policy information about availability of data

All manuscripts must include a data availability statement. This statement should provide the following information, where applicable:

- Accession codes, unique identifiers, or web links for publicly available datasets
- A description of any restrictions on data availability
- For clinical datasets or third party data, please ensure that the statement adheres to our policy

The non-image data used for analysis and figures are provided as Source Data files. The time-series image data for Swiss site generated in this study have been deposited in a Dryad repository [DOI: 10.5061/dryad.1g1jwsv11]. The time-series image data for Japanese site generated in this study as well as the labelling data for image analysis used in this study are available in a Dryad repository [DOI: 10.5061/dryad.h70rxwdnk]. Note that each Dryad datasets is large, nearly 250 GB, and that visiting the Dryad links automatically starts the downloading process which can be canceled and resumed later.

Human research participants

Policy information about studies involving human research participants and Sex and Gender in Research.

Reporting on sex and gender	N/A
Population characteristics	N/A
Recruitment	N/A
Ethics oversight	N/A

Note that full information on the approval of the study protocol must also be provided in the manuscript.

Field-specific reporting

Please select the one below that is the best fit for your research. If you are not sure, read the appropriate sections before making your selection.

☒ Life sciences ☐ Behavioural & social sciences ☐ Ecological, evolutionary & environmental sciences

For a reference copy of the document with all sections, see nature.com/documents/nr-reporting-summary-flat.pdf

Life sciences study design

All studies must disclose on these points even when the disclosure is negative.

Sample size	No sample size calculation was performed.
Data exclusions	For color and anthocyanin measurement, data from 20200121 were excluded because the target plants were hard to recognize due to strong light. One sample with relative anthocyanin value of > 0.07 was excluded as an outlier as the value was an order larger than the rest. Four samples were excluded because of incomplete record. For analysis based on the time-series images, a part of the data were excluded according to the criteria described in the manuscript. Exclusion criteria were not pre-established.
Replication	For color and anthocyanin measurement and time-series image analyses, we defined each individual as a biological replicate. For the former, we had eight replicates per time point per genotype with rare cases with slightly less than eight. We planted 20 replicates per genotype for time-series image analysis. The number of replicates were less at some time points, but we measured at least three replicates per time point per genotype. All of the results were successfully replicated.
Randomization	Plants for color and anthocyanin measurement were chosen from a different row in the garden for each time point. Plants for time-series image analysis were planted in a randomized complete block design.
Blinding	This study does not include data based on human senses or judgment. We consider blinding is not required in such a case.

Reporting for specific materials, systems and methods

We require information from authors about some types of materials, experimental systems and methods used in many studies. Here, indicate whether each material, system or method listed is relevant to your study. If you are not sure if a list item applies to your research, read the appropriate section before selecting a response.

Materials & experimental systems

n/a	Involved in the study
☒	☐ Antibodies
☒	☐ Eukaryotic cell lines
☒	☐ Palaeontology and archaeology
☐	☒ Animals and other organisms
☒	☐ Clinical data
☒	☐ Dual use research of concern

Methods

n/a	Involved in the study
☒	☐ ChIP-seq
☒	☐ Flow cytometry
☒	☐ MRI-based neuroimaging

Animals and other research organisms

Policy information about studies involving animals; ARRIVE guidelines recommended for reporting animal research, and Sex and Gender in Research

Laboratory animals	The study did not involve laboratory animals.
Wild animals	The study did not involve wild animals.
Reporting on sex	The study was restricted to using plants and is not relevant to reporting on sex.
Field-collected samples	The study did not involve samples collected from the field. We observed plants planted and grown in the common gardens.
Ethics oversight	The study was restricted to using plants and is not relevant to ethics oversight.

Note that full information on the approval of the study protocol must also be provided in the manuscript.
