# Supplementary material for: Seasonal pigment fluctuation in diploid and polyploid Arabidopsis revealed by machine learning-based phenotyping method PlantServation
Source: Nat Commun. 2023 Sep 22;14:5792. doi: 10.1038/s41467-023-41260-3 (PMC10517152; doi:10.1038/s41467-023-41260-3)
Supplement: Supplementary file 8 — Description of Additional Supplementary Files [file 41467_2023_41260_MOESM8_ESM.pdf]

Description of Additional Supplementary Files

File Name: Supplementary Movie 1
Description: Time-series compilation of the segmented plant area
Images of *Arabidopsis thaliana* (COL) from camera 04 in the Swiss site during the 2018–2019 season (yr2). The cyan lines indicate the segmented areas. The corresponding RGB values are shown in the images. Anomalous data were removed as described in the Methods section, except for snow cover that was retained to facilitate the visual recognition of the effect of snow on the target plants.

File Name: Supplementary Movie 2
Description: Time-series compilation of the segmented plant area
Images of Japanese *Arabidopsis kamchatica* (MAG) from camera 04 in the Swiss site during the 2018–2019 season (yr2). The cyan lines indicate the segmented areas. The corresponding RGB values are shown in the images. Anomalous data were removed as described in the Methods section, except for snow cover that was retained to facilitate the visual recognition of the effect of snow on the target plants.

File Name: Supplementary Movie 3
Description: Time-series compilation of the segmented plant area
Images of synthetic *Arabidopsis kamchatica* (RS7) from camera 04 in the Swiss site during the 2018–2019 season (yr2). The cyan lines indicate the segmented areas. The corresponding RGB values are shown in the images. Anomalous data were removed as described in the Methods section, except for snow cover that was retained to facilitate the visual recognition of the effect of snow on the target plants.

File Name: Supplementary Data 1
Description: The scripts used for analyzing non-image data and producing figures in the manuscript.
